# Penalized regression and model selection methods for polygenic scores on summary statistics

**DOI:** 10.1371/journal.pcbi.1008271

**Published:** 2020-10-01

**Authors:** Jack Pattee, Wei Pan

**Affiliations:** Division of Biostatistics, School of Public Health, University of Minnesota, Minneapolis, Minnesota; HONG KONG

## Abstract

Polygenic scores quantify the genetic risk associated with a given phenotype and are widely used to predict the risk of complex diseases. There has been recent interest in developing methods to construct polygenic risk scores using summary statistic data. We propose a method to construct polygenic risk scores via penalized regression using summary statistic data and publicly available reference data. Our method bears similarity to existing method LassoSum, extending their framework to the Truncated Lasso Penalty (TLP) and the elastic net. We show via simulation and real data application that the TLP improves predictive accuracy as compared to the LASSO while imposing additional sparsity where appropriate. To facilitate model selection in the absence of validation data, we propose methods for estimating model fitting criteria AIC and BIC. These methods approximate the AIC and BIC in the case where we have a polygenic risk score estimated on summary statistic data and no validation data. Additionally, we propose the so-called quasi-correlation metric, which quantifies the predictive accuracy of a polygenic risk score applied to out-of-sample data for which we have only summary statistic information. In total, these methods facilitate estimation and model selection of polygenic risk scores on summary statistic data, and the application of these polygenic risk scores to out-of-sample data for which we have only summary statistic information. We demonstrate the utility of these methods by applying them to GWA studies of lipids, height, and lung cancer.

## Author summary

Polygenic risk scores use genetic data to predict the genetic risk associated with a given phenotype. Often, due to privacy concerns, genetic data is provided in a limited format called summary statistics. This means that we have limited data with which to estimate polygenic risk scores and cannot apply many standard modelling techniques. We provide novel methods for the estimation of polygenic risk scores via penalized regression using summary statistics, and make software available to do this estimation. We also provide novel methods for model selection and the assessment of model performance in the summary statistic framework. In total, this enables us to use summary statistic data to estimate polygenic risk scores, select a polygenic risk score from among a set of candidate models, and assess the performance of these models. This allows us to leverage summary statistic data to better understand genetic risk. We establish the usefulness of our novel methods via simulation, and apply them to genetic analyses of height, blood lipid levels, and lung cancer.

This is a *PLOS Computational Biology* Methods paper.

## Introduction

The polygenic model of inheritance predicts that the genetic basis of complex phenotypes consists of small effects from thousands of genetic variants. Genome-wide association studies (GWAS) have affirmed this model, identifying many genetic variants that are associated with complex traits [1]. However, marginally associated markers explain only a limited proportion of the heritability of many traits [2]. Polygenic risk scores, defined as a linear combination of individual SNP effects, have been used to quantify the genetic component of some complex phenotypes. Polygenic risk scores estimated from GWAS have been useful for predicting some clinical phenotypes [3–5]. Polygenic risk scores have also been used to infer the genetic architecture of complex traits [6, 7]. The simple polygenic risk score is obtained by summing marginal genetic effects across all SNPs. Extensions on this method include thresholding [8], in which SNPs with marginal p-values below a certain cutoff point are excluded, and pruning and thresholding, which combines thresholding with the exclusion of highly correlated SNPs via pruning [9]. These methods use only marginal effect size estimates, and do not attempt to construct a joint model that estimates effect sizes under linkage disequilibrium. Thus, it can be said that they do not attempt to model the true structure of the genetic effects. We propose a method for constructing polygenic risk scores that integrates marginal effect size estimates with publicly available reference panel data, which is used to estimate linkage disequilibrium. By estimating effect sizes under linkage disequilibrium, we more closely model the true structure of the genetic effects. This allows us to capture more of the genetic heritability, as shown via simulation and application to real data.

Popular methods LDPred [10], LassoSum [11], and JAMPred [12] estimate joint models that account for linkage disequilibrium. Recently published methods in this area include PRS-CS [13] and SBayesR [14]. Other methods, such as EBPRS [15], leverage the available GWAS data to estimate a distribution of SNP effect sizes that is leveraged to adjust the marginal SNP effects. These methods do not necessitate individual level data. They use publicly available reference data and published summary statistics from GWAS. This is important because often the published results from a GWAS do not include individual level information. Our software implements new penalized regression methods for estimating polygenic risk scores that model linkage disequilibrium. Given a reference panel and marginal SNP effects, the software constructs a joint penalized regression model. We extend upon the work of Shin et al [11], who propose using the LASSO penalty, to other penalties: namely the truncated LASSO penalty [16] and the elastic net penalty [17]. These penalties have some theoretical benefits as compared to the LASSO penalty; the TLP may induce more sparsity when the truth is sparse and produce less biased estimates, while the elastic net may handle correlated covariates more stably. The TLP also has application for valid inference that may be useful [18]. We call these methods TlpSum and ElastSum, respectively.

Additionally, we describe some criteria that can be used for model selection in the case where we do not have access to validation data. In an application where we have access to validation data, we may select the model that maximizes the correlation between the estimated polygenic risk score and the validation phenotype. In the case where we don’t have access to validation data, we may still want to perform model selection on a set of candidate polygenic risk scores. Our methodology approximates the model fitting criteria AIC and BIC in the situation where we do not have individual level data. These methods, so called ‘pseudo AIC’ and ‘pseudo BIC’, approximate the AIC and BIC criteria for an estimated polygenic risk score given GWAS summary statistics and a reference panel. These methods extend upon the existing model selection criterion pseudovalidation [11]. Pseudovalidation controls model degrees of freedom by weighting SNPs by their local false discovery rate. This method is somewhat ad hoc, and local FDR estimation may not perform reliably when we don’t have a dense set of summary statistics available. This is often the case with published summary statistics, which may only include SNPs above some marginal significance threshold. Pseudo AIC and pseudo BIC leverage the well established theory of AIC and BIC to impose a penalty on degrees of freedom. This leads the pseudo AIC and BIC to select sparser models that more accurately represent the truth, as demonstrated via simulation study. We also show that pseudo BIC and pseudo AIC select models with better predictive performance on out-of-sample data in certain simulation settings and in application to a large GWAS of blood lipid levels.

Lastly, we propose a metric for assessing the predictive accuracy of a polygenic risk score in the case where we have only summary statistic information on our out-of-sample data. We call this metric ‘quasi-correlation’. Given an estimated polygenic risk score and a reference panel, this method allows us to estimate the predictive *r*^2^ of the polygenic risk score as applied to an out-of-sample dataset comprised of summary statistics. Thus, we can determine which model fits best on out-of-sample data given a candidate set of polygenic risk scores. This enables us to select a validated polygenic risk score ready for use on other data. These methods allow us to use published summary statistic data of large sample size to assess the predictive accuracy of polygenic risk scores, broadening the scope of application. We demonstrate the utility of these methods by applying them to large GWAS summary statistic data on lipids. Applications to lung cancer and height are located in **Section F in** S1 Text and **Section G in** S1 Text, respectively.

The central aim of this paper is to assess the predictive performance of the methods for polygenic risk score estimation and corresponding model selection. We demonstrate that TlpSum and ElastSum often perform similarly to LassoSum as measured by predictive accuracy, but outperform LassoSum when applied to data with substantial allelic heterogeneity. We show that our proposed model selection methods, pseudo AIC and pseudo BIC, select models with better predictive performance on out-of-sample data than pseudovalidation in certain applications. A secondary but relevant concern is the characterization of the fitted models in terms of sparsity. Given a set of models with similar predictive performance on out-of-sample data, it is often desirable to select the most parsimonious model, which is the so-called principle of parsimony. A more parsimonious model that maintains good predictive performance better facilitates interpretation and certain applications of polygenic risk scoring. One useful example is the application of polygenic risk scoring to two-stage least squares regression for causal inference [19]. In this type of applications, overparameterized PRS models may contain substantial pleiotropic effects, making causal inference difficult with violated modeling assumptions. In this paper, we demonstrate via simulation that TlpSum, pseudo AIC, and pseudo BIC impose additional sparsity on their selected models. We discuss the results with respect to the primary aim of prediction, and characterize the selected models to possibly explain the performance difference among the various methods.

An implementation of the methods described in this paper is provided in our R package ‘penRegSum’, located at https://github.com/jpattee/penRegSum. This package directly interfaces with PLINK for ease of computation [20, 21]. We note that the authors of the LassoSum package [11] provide extensive functionality for the estimation and selection of polygenic scores on summary data. Our package can be considered an extension on their work that is best used in conjunction with their package. TlpSum demonstrates reasonable computational cost as compared to other methods for polygenic risk score estimation on summary statistics, as described in **Section H in** S1 Text.

## Methods

### Penalized regression with summary statistics

Consider that we have a linear regression model

$$\begin{array}{c} y=X\beta +\epsilon \end{array}$$ (1)

with **X** denoting an *n* × *p* design matrix, **y** denoting a vector of observed outcomes, and ***ϵ*** ∼ *MVN*(**0**, *σ*^2^**I**) for some *σ*^2^. Ordinary least squares estimates are obtained by minimizing the sum of squared errors

$$\begin{array}{c} f\left(\beta \right)=SSE={\left(y-X\beta \right)}^{T}\left(y-X\beta \right) \end{array}$$ (2)

In the case where *p* is large and ***β*** may be sparse, penalized regression models can be useful. Penalized regression models introduce a penalty term to the objective function. This penalty term is typically a function of ***β***, and is denoted *J*(***β***). Additionally, consider now that **y** is a standardized response vector, and **X** is a standardized design matrix. This yields the following objective function:

$$\begin{array}{c} f\left(\beta \right)={\left(y-X\beta \right)}^{T}\left(y-X\beta \right)+J\left(\beta \right)=y^{T}y+\beta^{T}X^{T}X\beta -2\beta^{T}X^{T}y+J\left(\beta \right). \end{array}$$

Shin et al [11] note that, given some approximations, penalized regression can be used to estimate polygenic risk scores in the case where only summary statistics are available. Consider that we have two separate datasets: one of summary statistics, and one of reference data. We use the summary statistic data to estimate univariate SNP effects, and the reference data to estimate the correlation matrix of the SNPs. Let us denote the standardized phenotype vector from the summary statistic data divided by $\sqrt{N}$ (the sample size of the summary statistic data) as **y**_*s*_. Let us denote the standardized summary statistic design matrix as **X**_*s*_. Let us denote the standardized SNP reference data as **X**_*r*_. We can now define the quantity

$$\begin{array}{c} r=X_{s}^{T}y_{s}, \end{array}$$

where **r** represents the SNP-wise correlation between the SNPs and the phenotype in the summary statistic data. We also define **R**, which is the correlation matrix as estimated from the reference data as

$$\begin{array}{c} R=X_{r}^{T}X_{r}. \end{array}$$

Given these approximations, we can now define an objective function for the estimation of polygenic risk scores using summary statistic data. That objective function is as follows:

$$\begin{array}{c} f\left(\beta \right)=y^{T}y+\beta^{T}R\beta -2\beta^{T}r+J\left(\beta \right). \end{array}$$

Shin et al. note that this is no longer strictly a penalized regression problem, due to the use of two different design matrices **X**_*r*_ and **X**_*s*_. This may lead to unstable and non-unique solutions. They propose regularizing **R** as

$$\begin{array}{c} R_{s}=\left(1-s\right)X_{r}^{T}X_{r}+sI \end{array}$$ (3)

for some 0 < *s* < 1. This regularization ensures that we have an objective function in the form of a LASSO problem, as proven in previous literature [11]. Substituting **R**_*s*_ for **R** yields the following tractable objective function:

$$\begin{array}{c} f\left(\beta \right)=y^{T}y+\beta^{T}R_{s}\beta -2\beta^{T}r+J\left(\beta \right). \end{array}$$ (4)

We now turn our attention to the penalty term *J*(***β***). Shin et al propose using the LASSO penalty, which is a popular penalized regression method for high dimensional problems. The LASSO induces sparsity in ***β***, performing parameter selection and estimation simultaneously. In LASSO, the penalty term is the L1 penalty, ie *J*(***β***) = λ∥*β*∥_1_ = λ∑_*i*_|*β*_*i*_|, where λ is a tuning parameter selected via a model selection method. The LASSO tends to bias parameter estimates towards zero in a uniform manner. This causes biased effect size estimates. To mitigate bias issues, we propose the use of the Truncated Lasso Penalty, or the TLP [16]. The TLP can be expressed as follows: *J*(***β***, *τ*) = λ∑_*i*_ *min*(|*β*_*i*_|, *τ*), where λ and *τ* are tuning parameters determined via model selection. The TLP does not penalize effect size estimates above some threshold *τ*, which may decrease bias. Additionally, we propose the use of the elastic net penalty [17], namely *J*(***β***) = *α*λ∥***β***∥_1_ + (1 − *α*)λ∥***β***∥^2^. This method has some advantages of the LASSO while retaining some advantages of ridge regression, such as stable estimation of highly correlated covariates. The application of the elastic net penalty to summary statistics is called ElastSum. We note that the use of the regularized covariance matrix approximates a sort of elastic net already. To see that, consider the expanded expression of Eq (4):

$$\begin{array}{c} f\left(\beta \right)=y^{T}y+\left(1-s\right)\beta^{T}R\beta +s\beta^{T}\beta -2\beta^{T}r+J\left(\beta \right). \end{array}$$ (5)

We note that the term *s* ***β***^*T*^***β*** approximates the L2 penalty. Given this, we are unsure of the utility of the elastic net penalty in many cases. We also note that this may affect the TLP estimates. If *s* > 0, the objective function will function somewhat like an elastic net, meaning the TLP may not induce its characteristic sparsity.

### Notes on application of penalized regression

If SNPs are in high linkage disequilibrium, then it may be difficult or impossible to arrive at stable estimates for ***β*** given the objective function (4). In this case, we advise performing LD clumping on the data prior to estimating a penalized regression model. LD clumping should prioritize SNPs in the target data where model performance is assessed. Even after clumping, it is often the case that convergence is impossible (or very slow) unless a sufficiently large value of *s* is chosen. The value of s chosen depends on the sparseness of the genetic signal. For phenotypes with a sparse genetic signal, choosing an *s* as small as 0 may work, and choosing *s* ∼ .1 should ensure good convergence and fairly sparse effect size estimates. For phenotypes with more dense signal, we recommend experimenting with larger values of *s*.

As Shin et al note [11], penalized regression generates effect size estimates that are not appropriately scaled. Considering that penalized regression is conducted on normalized data, we can say these estimates are scaled as correlations. If we want to use a polygenic risk score generated via penalized regression to estimate genetic risk, we need to appropriately scale our estimates. We have the following expression for the effect size estimates: ${\hat{\beta }}_{i}^{unstandardized}={\hat{\beta }}_{i}\frac{sd(y)}{sd(X_{i})}$, where *X*_*i*_ is column *i* in the reference panel **X**_*r*_, **y** is the phenotype vector, and ${\hat{\beta }}_{i}$ are the effect size estimates produced by the penalized regression; that is, those estimates minimizing objective function (4). Note that ${\hat{\beta }}_{i}^{unstandardized}$ is equivalent to the per-allele effect size.

Existing literature demonstrates that estimation by LD blocks improves the predictive performance of penalized regression methods applied to summary statistics [11]. We also recommend performing estimation by LD blocks, and do so in this paper unless otherwise specified. Supplementary **Section C in** S1 Text displays some simulation results estimated without LD blocks, and the modest loss in predictive power that ensues. We used LD blocks as defined by the LDetect method [22].

If some SNPs in the summary statistic data are not included in the reference data, we are not able to incorporate those SNPs into our objective function (5) as currently formulated. Simply excluding these SNPs from our analysis may result in information loss. A straightforward approach, as described by the authors of the LassoSum paper, is to treat these SNPs as though they are mutually independent. We take an identical approach here. We define ***β***_0_ as a subvector of ***β*** corresponding to those SNPs missing from the reference panel, and the corresponding submatrix **R**_0_ of **R** as containing all zero entries. Thus, we can reformulate the objective function as follows:

$$\begin{array}{c} f\left(\beta \right)=y^{T}y+\left(1-s\right)\beta^{T}R\beta +s\beta^{T}\beta -2\beta^{T}r+\left(1-s\right)\beta_{0}^{T}\beta_{0}+J\left(\beta \right) \end{array}$$

This approach to handling SNPs missing from the reference panel has been implemented in our R package.

We estimate penalized regression models on summary statistics via coordinate descent [23]. The details of this algorithm are located in **Section 1 in** S1 Text.

### Pseudo AIC / BIC

It may be the case that we do not have access to validation data for use in model selection. In this case, it is desirable to have a model selection technique to select tuning parameters that can be applied to summary statistic data. Shin et al propose the pseudovalidation method for this purpose [11]. Pseudovalidation approximates the correlation between the predicted phenotypes and the phenotypes from the summary statistic data. One drawback of their method is that it may tend to overfit the model, as the pseudovalidation criteria will tend to increase as parameters are added to the model. This is somewhat controlled for by their weighting of marginal p-values by local FDR, but this isn’t necessarily a rigorous approcah. We propose a method of estimating model fitting metrics AIC and BIC using only summary statistics and a reference panel. We believe these methods may select less overfit and therefore sparser models.

Suppose that we have trait **Y** measured on *N* subjects. We have data on *p* SNPs for each subject, giving us *N* × *p* design matrix **X**. Say **X**, **Y** are centered at zero. We assume model (1). Given this, we have the following likelihood function:

$$\begin{array}{c} L=\prod_{i=1}^{N}p\left(y_{i}|x_{i};\beta ,\sigma^{2}\right)\propto \sigma^{-N}\text{exp}\left[{\left(Y-X\beta \right)}^{\prime }\left(Y-X\beta \right)/\left(2\sigma^{2}\right)\right] \end{array}$$

and the following log-likelihood function:

$$\begin{array}{c} l=C-N*ln\sigma -\frac{1}{2\sigma^{2}}\left(Y^{\prime }Y-2\beta^{\prime }X^{\prime }Y+\beta^{\prime }X^{\prime }X\beta \right). \end{array}$$

C does not depend on the parameters, and so can be ignored.

Placing this problem in our summary statistic framework, we want to estimate **Y**′**Y**, **X**′**Y** and **X**′**X** from reference data and summary statistics. Suppose we have univariate summary statistics $\hat{\beta }={\left({\hat{\beta }}_{1},{\hat{\beta }}_{2},\ldots ,{\hat{\beta }}_{p}\right)}^{\prime }$ and corresponding variances $\hat{var}\left({\hat{\beta }}_{j}\right)$, which quantify the marginal associations between phenotype **y** and each of the *p* SNPs in design matrix **X**. We also have reference panel **X**_*r*_. Denote the variance of a given SNP *j*, as estimated from the reference panel, as ${\hat{s}}_{j}^{2}$. We define that there are *N* individuals in **X** and *n* individuals in **X**_*r*_. Because of the differing sample sizes, we want to compare quantities that have been normalized by sample size when estimating the log-likelihood. With this in mind, we define the following approximations:

$$\begin{array}{c} \frac{1}{N}\hat{X^{T}X}=\Sigma =\frac{1}{n}X_{r}^{T}X_{r}, \end{array}$$ (6)

$$\begin{array}{c} \frac{1}{N}\hat{Y^{\prime }Y}=\left(N*{\hat{s}}_{j}^{2}*\hat{var}\left({\hat{\beta }}_{j}\right)+{\hat{s}}_{j}^{2}*{\hat{\beta }}_{j}^{2}\right), \end{array}$$ (7)

$$\begin{array}{c} \frac{1}{N}\hat{X^{\prime }Y}=diag\left(\Sigma \right){\left({\hat{\beta }}_{1},{\hat{\beta }}_{2},\ldots ,{\hat{\beta }}_{p}\right)}^{\prime }={\left({\hat{s}}_{1}^{2}{\hat{\beta }}_{1},{\hat{s}}_{2}^{2}{\hat{\beta }}_{2},\ldots ,{\hat{s}}_{p}^{2}{\hat{\beta }}_{p}\right)}^{\prime }. \end{array}$$ (8)

Note that $diag\left(\Sigma \right)=\left({\hat{s}}_{1}^{2},\ldots ,{\hat{s}}_{p}^{2}\right)$. In practice, we advise taking some central tendency the expression for $\frac{1}{N}\hat{Y^{\prime }Y}$ across the *p* SNPs to obtain a more accurate approximation. We have found the median to work well.

We briefly justify approximations (6), (7), and (8) here. Expression (6) simply describes the approximation of the covariance matrix by a reference panel. Expression (7) can be derived as follows. Consider that, for single linear regression, $\hat{\beta_{i}}=\frac{\sum_{j=1}^{N}x_{ji}y_{j}}{N*s_{i}^{2}}$ and $\hat{var}\left({\hat{\beta }}_{i}\right)=\frac{\sum_{j=1}^{N}{\left(y_{j}-x_{ji}{\hat{\beta }}_{i}\right)}^{2}}{N^{2}*s_{i}^{2}}$. Thus we have: $\frac{1}{N}\hat{Y^{\prime }Y}=N*s_{i}^{2}*\frac{\sum_{j=1}^{N}{\left(y_{j}-x_{ji}{\hat{\beta }}_{i}\right)}^{2}}{N^{2}*s_{i}^{2}}+s_{i}^{2}*{\hat{\beta }}_{i}^{2}$. Expanding the squared term and using the fact that $\hat{\beta_{i}}=\frac{\sum_{j=1}^{N}x_{ji}y_{j}}{N*s_{i}^{2}}$, we have: $N*s_{i}^{2}*\frac{\sum_{j=1}^{N}{\left(y_{j}-x_{ji}{\hat{\beta }}_{i}\right)}^{2}}{N^{2}*s_{i}^{2}}=\frac{\sum_{j=1}^{N}{\left(y_{j}-x_{ji}{\hat{\beta }}_{i}\right)}^{2}}{N}=\frac{\sum_{j=1}^{N}y_{j}^{2}}{N}-2{\hat{\beta }}_{i}^{2}*s_{i}^{2}+{\hat{\beta }}_{i}^{2}*s_{i}^{2}$. Thus, we conclude that $\frac{1}{N}\hat{Y^{\prime }Y}=\frac{\sum_{j=1}^{N}y_{i}^{2}}{N}$. Now, we examine expression (8). Given that $\hat{\beta_{i}}=\frac{\sum_{j=1}^{N}x_{ji}y_{j}}{N*s_{i}^{2}}$, it is straightforward that $\frac{\sum_{j=1}^{N}x_{ji}y_{j}}{N}=s_{i}^{2}{\hat{\beta }}_{i}$. Expression (8) follows from this. Note that expressions (6), (7), and (8) have been derived assuming single linear regression. Given some mild assumptions and changes in interpretation, these expressions are still valid when summary statistics are estimated using multiple regression, i.e. in a GWAS that includes non-SNP covariates. Details are in **Section L in** S1 Text.

To estimate the log likelihood of a linear regression model, we must estimate the sum of squared errors (2). Additionally, we must estimate the residual variance ${\tilde{\sigma }}^{2}$. We estimate the SSE with the penalized regression estimates, which we denote ${\hat{\beta }}_{P}$. Note that these differ from the marginal effect size estimates $\hat{\beta }$. We estimate the residual variance with the ordinary least square estimates, denoted ${\hat{\beta }}_{OLSE}$.

To estimate ${\tilde{\sigma }}^{2}$, we use the ordinary least squares estimates

$$\begin{array}{c} {\hat{\beta }}_{OLSE}={\left(X^{\prime }X\right)}^{-1}X^{\prime }Y. \end{array}$$

For a linear regression, we have residual variance estimated as follows:

$$\begin{array}{c} {\tilde{\sigma }}^{2}=MSE=\frac{{\left(Y-X\beta \right)}^{\prime }\left(Y-X\beta \right)}{N-q} \end{array}$$

where *q* is the degrees of freedom. In the case of linear regression, this is equivalent to the number of parameters. We substitute $\beta ={\hat{\beta }}_{OLSE}$ to yield

$$\begin{array}{c} {\tilde{\sigma }}^{2}=\frac{Y^{\prime }Y-Y^{\prime }X{\left(X^{\prime }X\right)}^{-1}X^{\prime }Y}{N-q}. \end{array}$$

The above expression is tractable given our substitutions. Because our approximations (6), (7), and (8) have been normalized by sample size before comparison, the value we get from direct comparison is equivalent to a so-called “average SSE”, and must be multiplied by the sample size *N*. This yields the following expression:

$$\begin{array}{c} {\hat{\tilde{\sigma }}}^{2}=\frac{1}{N-q}\left[\frac{1}{N}\hat{Y^{\prime }Y}-{\left(\frac{1}{N}\hat{X^{\prime }Y}\right)}^{T}{\left(\frac{1}{N}\hat{X^{\prime }X}\right)}^{-1}\frac{1}{N}\hat{X^{\prime }Y}\right]\times N. \end{array}$$ (9)

We demonstrate the effectiveness of estimator ${\hat{\tilde{\sigma }}}^{2}$ via simulation in **Section E in** S1 Text, and show that it behaves well as compared to some other plausible estimators. To calculate the SSE based on some set of estimates ${\hat{\beta }}_{P}$, we substitute our approximations (6), (7) and (8) and the penalized regression estimates into the following expanded expression for SSE:

$$\begin{array}{c} SSE=Y^{\prime }Y-2\beta^{\prime }X^{\prime }Y+\beta^{\prime }X^{\prime }X\beta . \end{array}$$

This yields the following expression:

$$\begin{array}{c} \hat{SSE}=\left(\frac{1}{N}\hat{Y^{\prime }Y}-2{\hat{\beta }}_{P}^{{}^{\prime }}\frac{1}{N}\hat{X^{\prime }Y}+{\hat{\beta }}_{P}^{{}^{\prime }}\frac{1}{N}\hat{X^{\prime }X}{\hat{\beta }}_{P}\right)\times N. \end{array}$$ (10)

Note that, as in the estimation of ${\tilde{\sigma }}^{2}$, we multiply by the expression for SSE by *N* because all of the terms in the expression are normalized.

Given this, we can express our log-likelihood, as estimated from the reference panel and summary statistics, as follows:

$$\begin{array}{c} l=-\frac{1}{2{\hat{\tilde{\sigma }}}^{2}}\hat{SSE}. \end{array}$$

Consider that we have omitted constants from the above expression that do not affect the relative values of the pseudo AIC / BIC. Given the log-likelihood, we can construct the pseudo AIC and BIC as follows, which mirrors existing literature on AIC and BIC for penalized regression [24]:

$$\begin{array}{c} AIC=2k-2l, \end{array}$$

$$\begin{array}{c} BIC=ln(N)*k-2l. \end{array}$$

Where *k* is the degrees of freedom of the model, and *l* is the log-likelihood. Since our penalized regression models can be thought of as a form of elastic net, we use the degrees of freedom of the ridge regression model, calculated as $df\left(\lambda ,s\right)=tr[{\left(X_{r}^{{}^{\prime }}X_{r}+\lambda sI\right)}^{-1}\left(X_{r}^{{}^{\prime }}X_{r}\right)]$. If this is too intensive to calculate for data with a large number of parameters *p*, we can also use the degrees of freedom for the LASSO model, which is simply the number of nonzero parameter estimates.

### Notes on the application of pseudo AIC / BIC

In the case where we have a binary phenotype, the univariate effect size estimates are typically obtained via logistic regression. We note that the theory on pseudo AIC / BIC applies only to linear regression, and is intractable for logistic regression. Thus, we want to convert the univariate logistic regression estimates to univariate linear regression estimates $\hat{\beta }$.

Consider that we have some binary vector of phenotypes **y** and a centered design matrix **X** where each column is a SNP. We start with the condition that linear and logistic regression should give similar results; that is, for any entry *x*_*ij*_, *π* = *p*(*y*_*i*_ = 1|*x*_*ij*_) is approximately equal under linear and logistic regression. Let us denote the logistic regression estimates as ${\left({\hat{b}}_{0},{\hat{b}}_{1}\right)}^{\prime }$, and the linear regression estimates as ${\left({\hat{\beta }}_{0},{\hat{\beta }}_{1}\right)}^{\prime }$. Thus, we have:

$$\begin{array}{c} {\hat{\beta }}_{0}+{\hat{\beta }}_{1}x_{ij}=\frac{1}{1+e^{-({\hat{b}}_{0}+{\hat{b}}_{1}x_{ij})}}. \end{array}$$

Given that these two expressions are equivalent, we know that the terms comprising their respective Taylor expansions are also equivalent. Taking the first term of the Taylor expansion of each expression and equating them, we get the get the following equivalence:

$$\begin{array}{c} {\hat{\beta }}_{0}=\frac{1}{1+e^{-{\hat{b}}_{0}}}. \end{array}$$

Taking the second term of the Taylor expansion for each expression and equating them, we get the equivalence

$$\begin{array}{c} {\hat{\beta }}_{1}x_{ij}=\frac{e^{-{\hat{b}}_{0}}}{{\left(1+e^{-{\hat{b}}_{0}}\right)}^{2}}{\hat{b}}_{1}x_{ij}, \end{array}$$

from which we have, straightforwardly,

$$\begin{array}{c} {\hat{\beta }}_{1}=\frac{e^{-{\hat{b}}_{0}}}{{\left(1+e^{-{\hat{b}}_{0}}\right)}^{2}}{\hat{b}}_{1}. \end{array}$$

Note that $e^{-b_{0}}=\frac{p(Y=0)}{p(Y=1)}$, which is easily obtainable. It is simply the ratio of controls to cases in the phenotype vector **y**. This method will work best when *b*_1_ is small, and thus the slope of the estimated logistic function is shallow. This is almost always the case in GWAS applications given the small effect sizes of individual SNPs, so this approximation should hold.

We also need to approximate the standard error of the linear regression estimates. A derivation with a general formula and some discussion is contained in **Section I in** S1 Text. Following this derivation, we get the expression

$$\begin{array}{c} \hat{var}\left({\hat{\beta }}_{1}\right)={\left(\frac{e^{-{\hat{b}}_{0}}}{{\left(1+e^{-{\hat{b}}_{0}}\right)}^{2}}\right)}^{2}var\left({\hat{b}}_{1}\right), \end{array}$$

and thus

$$\begin{array}{c} \hat{SE}\left({\hat{\beta }}_{1}\right)=(\frac{e^{-{\hat{b}}_{0}}}{{\left(1+e^{-{\hat{b}}_{0}}\right)}^{2}})se\left({\hat{b}}_{1}\right). \end{array}$$

We assume the standard error of the logistic regression estimate $SE({\hat{b}}_{1})$ is contained in the summary statistic information, making this calculation straightforward. An application of our pseudo AIC/BIC methodology to binary lung cancer data is described in **Section F in** S1 Text.

Another issue is the selection of the degrees of freedom *q* in the calculation of the OLSE-based ${\hat{\tilde{\sigma }}}^{2}$. In the case where *p* > *N*, we cannot include all univariate summary statistics in our estimation of ${\hat{\tilde{\sigma }}}^{2}$. We propose using pruning and thresholding to determine a set of independent and moderately associated SNPs, and using this set for the calculation of ${\hat{\tilde{\sigma }}}^{2}$. Some experimentation has shown the estimation of ${\hat{\tilde{\sigma }}}^{2}$ to be relatively invariant across reasonable choices of a SNP set.

When calculating $\hat{SSE}$ and ${\hat{\tilde{\sigma }}}^{2}$ in practice, we found it useful to regularize the estimated covariance matrix. When estimating $\hat{SSE}$ as described in (10) and ${\hat{\tilde{\sigma }}}^{2}$ as described in (9), we make the substitution described below. Note that this bears some similarity to the regularization we describe in Eq (3) with *s* = .2, although it is not identical:

$$\begin{array}{c} \frac{1}{n}\hat{X^{T}X}=\frac{1}{n}X_{r}^{T}X_{r}+.2I. \end{array}$$

In our experience, when estimating penalized regression models via summary statistics and pseudo AIC / BIC via summary statistics on the same data, it is crucial not to reuse the same reference panel for the calculation of the polygenic risk scores and the calculation of the pseudo AIC / BIC. Doing so leads to a sort of overfitting issue, and will badly degrade the performance of the pseudo AIC / BIC. In practice, this can be avoided by splitting the reference panel in half, and using one half for the estimation of polygenic risk scores and the other half for the estimation of model fitting metrics.

As in the estimation of polygenic risk scores via penalized regression, we recommend estimating the pseudo AIC / BIC by independent LD blocks [22]. This is relevant to the covariance matrix $\frac{1}{n}\hat{X^{T}X}$ as estimated from the reference panel. All estimation of pseudo AIC / BIC in this paper was done by LD blocks unless otherwise noted.

### Quasi-correlation

The so-called quasi-correlation is a model-fitting metric that can be used to evaluate the performance of a polygenic risk score on out-of-sample data for which we have only summary statistics. It similar to existing method SummaryAUC [25], except that quasi-correlation is relevant to continuous phenotype data. The quasi-correlation estimates the true correlation. Because the correlation between a polygenic risk score and a validation phenotype is frequently used for model selection of polygenic risk scores, we can apply the quasi-correlation for model selection purposes as well.

Now, we describe the scenario when application of quasi-correlation is useful. In this scenario, we have three datasets. Firstly, we have the ‘training’ dataset, with centered design matrix denoted **X** and centered phenotype denoted **Y**. Using some method, such as penalized regression, we estimate a polygenic risk score. We denote this ${\hat{\beta }}^{P}={\left({\hat{\beta }}_{1}^{P},\ldots ,{\hat{\beta }}_{p}^{P}\right)}^{\prime }$. Secondly, we have the reference panel, denoted **X**_*r*_. That is, **X**_*r*_ is some centered matrix with columns corresponding to the same SNPs as those in **X**. We also use **X**_*r*_ to estimate the variances of the SNPs. Let’s denote this vector of estimated variances as $\left({\hat{s}}_{1}^{2},\ldots ,{\hat{s}}_{p}^{2}\right)$. Lastly, we have the ‘testing’ dataset, where we want to test the accuracy of our polygenic risk score ${\hat{\beta }}^{P}$. For this data, we have centered design matrix X_*_, centered phenotype Y_*_, and sample size *n*_*t*_. Using univariate linear regression, we estimate marginal effect sizes ${\hat{\beta }}_{*}$ for the testing data. We assume that we do not have access to either X_*_ or Y_*_, and only have ${\hat{\beta }}_{*}$.

We want to use our polygenic risk score ${\hat{\beta }}^{P}$ to predict phenotypes for the testing data. Then, we want to calculate the correlation between our estimated phenotypes on the testing data ${\hat{Y}}_{*}=X_{*}{\hat{\beta }}^{P}$ and the true phenotypes Y_*_. That is, we want to estimate the following quantity:

$$\begin{array}{c} cor\left(Y_{*},{\hat{Y}}_{*}\right)=\frac{\frac{1}{n_{t}}\sum_{i}Y_{i}^{*}{\hat{Y}}_{i}^{*}-\left(\frac{1}{n_{t}}\sum_{i}Y_{i}^{*}\right)\left(\frac{1}{n_{t}}\sum_{i}{\hat{Y}}_{i}^{*}\right)}{\sqrt{var\left(Y_{*}\right)var\left({\hat{Y}}_{*}\right)}}. \end{array}$$

We note that $(\frac{1}{n_{t}}\sum_{i}Y_{i}^{*})=0$, because we assume a centered Y_*_. Thus, we have the following expression:

$$\begin{array}{c} cor\left(Y_{*},{\hat{Y}}_{*}\right)=\frac{\frac{1}{n_{t}}\sum_{i}Y_{i}^{*}{\hat{Y}}_{i}^{*}}{\sqrt{var\left(Y_{*}\right)var\left({\hat{Y}}_{*}\right)}}. \end{array}$$

This is still not tractable, given that we cannot calculate ${\hat{Y}}_{*}$ directly because we don’t have access to X_*_. To obviate this, we make the following observation:

$$\begin{array}{c} \frac{1}{n_{t}}\sum_{i}Y_{i}^{*}{\hat{Y}}_{i}^{*}=\frac{1}{n_{t}}Y_{*}^{T}{\hat{Y}}_{*}=\frac{1}{n_{t}}Y_{*}^{T}X_{*}{\hat{\beta }}^{P}=\frac{1}{n_{t}}{\left(X_{*}^{T}Y_{*}\right)}^{T}{\hat{\beta }}^{P}=\sum_{j=1}^{p}{\hat{s}}_{j}^{2}{\hat{\beta }}_{j}^{*}{\hat{\beta }}_{j}^{P}. \end{array}$$

Now, we must find a way to estimate $var({\hat{Y}}_{*})$. To show the derivation, we introduce the notation that $X_{i}^{*T}$ is a transposed column vector corresponding to a row of X_*_. We then have

$$\begin{array}{c} var\left(\hat{Y_{*}}\right)=\frac{1}{n_{t}}\sum_{i}[{\left(X_{i}^{*T}{\hat{\beta }}^{P}\right)}^{2}]-{\left[\frac{1}{n_{t}}\sum_{i}\left(X_{i}^{*T}{\hat{\beta }}^{P}\right)\right]}^{2}=A-B. \end{array}$$

Now, we investigate terms *A* and *B*:

$$\begin{array}{c} A=\frac{1}{n_{t}}\sum_{i}{\left({\hat{\beta }}^{P}\right)}^{T}X_{i}^{*}X_{i}^{*T}{\hat{\beta }}^{P}={\left({\hat{\beta }}^{P}\right)}^{T}\left(\frac{1}{n_{t}}\sum_{i}X_{i}^{*}X_{i}^{*T}\right){\hat{\beta }}^{P}={\left({\hat{\beta }}^{P}\right)}^{T}X_{r}^{T}X_{r}{\hat{\beta }}^{P}, \end{array}$$

$$\begin{array}{c} B={\left[\frac{1}{n_{t}}\sum_{i}\left(X_{i}^{*T}{\hat{\beta }}^{P}\right)\right]}^{2}={\left(X_{*}{\hat{\beta }}^{P}\right)}^{2}=0. \end{array}$$

Finally, we must estimate *var*(Y_*_). We note that we can approximate the variance of a centered phenotype using summary statistics via Eq (7). As in the estimation of pseudo AIC / BIC, we suggest taking some measure of central tendency of the *p* estimates of $\hat{var}\left(Y_{*}\right)$, such as the median. Given these approximations, we can now define the quasi-correlation in a usable form:

$$\begin{array}{c} quasiCor\left(Y_{*},{\hat{Y}}_{*}\right)=\frac{\sum_{i}{\hat{s}}_{i}^{2}{\hat{\beta }}_{i}^{*}{\hat{\beta }}_{i}^{P}}{\sqrt{\hat{var}\left(Y_{*}\right){\left({\hat{\beta }}^{P}\right)}^{T}X_{r}^{T}X_{r}{\hat{\beta }}^{P}}}. \end{array}$$

## Results

### Simulation study for penalized regression

Here we show the effectiveness of penalized regression in predicting quantitative phenotypes in a simulation scenario. We show the accuracy of penalized regression compared to results from similar methods LDPred [10], LDPred-Inf, pruned and thresholded (P+T) polygenic risk scoring, and simple polygenic risk scoring.

We simulated quantitative phenotypes using data from the Wellcome Trust Case Control Consortium, or WTCCC [26]. We conducted three simulations. One simulation used genotype data from chromosomes 1 and 4 (∼ 30, 000 SNPs), which we will call simulation 1. The second simulation used genotype data from chromosomes 1, 2, 3 and 4 (∼ 61, 000 SNPs), which we will call simulation 2. The third simulation used SNPs from all chromosomes (∼ 230, 000 SNPs), which we call simulation 3. The ratio of sample size of the training data to the number of SNPs has been shown to affect the predictive performance of polygenic risk scoring in previous literature [10]; this ratio differentiates simulation 1, 2, and 3. The data was comprised of 12,479 individuals. The data were split into three sets: training, which consisted of 6240 individuals, tuning, which consisted of 3119 individuals, and testing, which consisted of 3120 individuals. We pruned SNPs such that no two included SNPs were in linkage disequilibrium higher than 0.9 in order to ensure convergence. In practice, one can perform LD clumping to ensure that no two SNPs are in LD higher than 0.9. We additionally removed all ambiguous SNPs (A/T, C/G), and all SNPs with *MAF* < .01.

We simulated SNP effect sizes from the point normal model:

$$\begin{array}{c} \beta_{j}{∼}_{iid}\{\begin{array}{c} N(0,\frac{h^{2}}{Mp}),\;\text{with}\;\text{probability}\;\text{p}\\ 0,\;\text{with}\;\text{probability}\;\text{1-p} \end{array} \end{array}$$

Where *h*^2^ is the SNP-based heritability of the disease (0.5 in our simulation), *M* is the number of SNPs, and *p* is the fraction of causal SNPs. We used the following values of *p* in our simulation: *p* = 0.1, *p* = 0.01, *p* = 0.001, *p* = .0005. Simulation 1 excluded the case where *p* = .0005, and simulation 3 excluded the case where *p* = .1. We used the SNP effects to generate quantitative phenotypes under the additive model of genetic effects. Using the simulated phenotypes and the training data, we calculated summary statistics. We used the summary statistics from the training data and LD information from the tuning data to estimate penalized regression models, using LassoSum, TlpSum, and ElastSum. With these penalized regression estimates, we generated predicted phenotypes for the tuning data set, and selected tuning parameter values that optimized the prediction *r*^2^. We then calculated predicted phenotypes for the test data, using the optimized tuning parameter values. We then report the predictive *r*^2^ of the testing data. We performed 20 replications for each method at each value of *p*.

**Fig 1:**
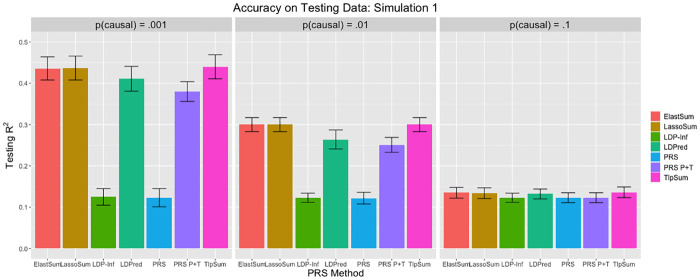
Prediction *r*^2^ values for simulation 1. Error bars represent standard deviation for the *r*^2^ value across 20 replications.

**Fig 2:**
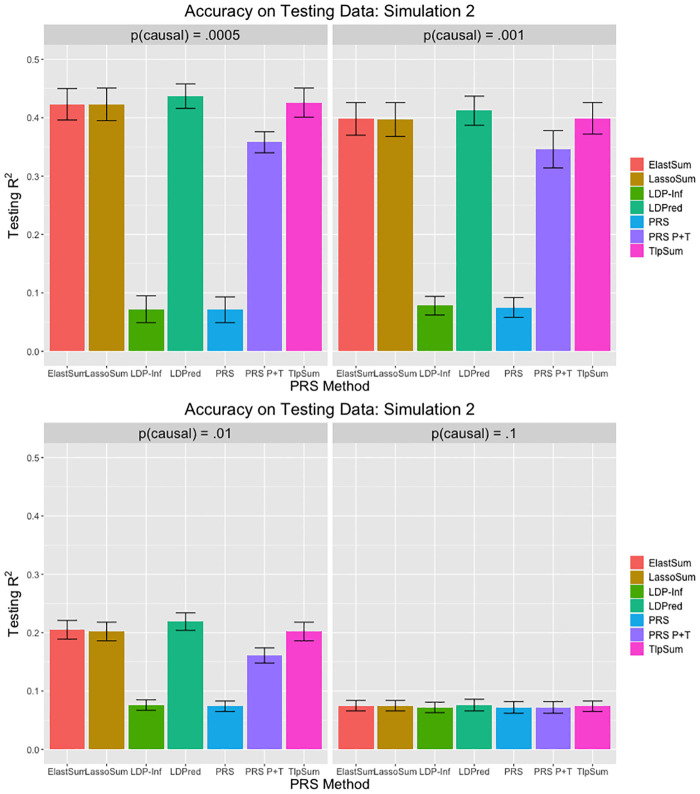
Prediction *r*^2^ values for simulation 2. Error bars represent standard deviation for the *r*^2^ value across 20 replications.

**Fig 3:**
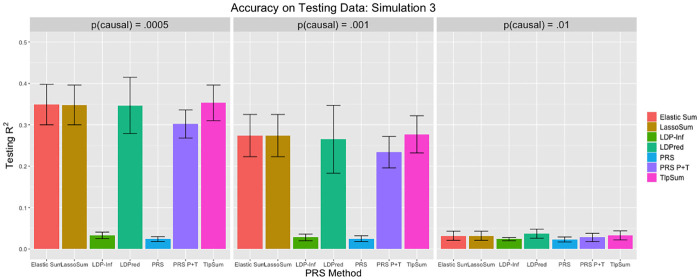
Prediction *r*^2^ values for simulation 3. Error bars represent standard deviation for the *r*^2^ value across 20 replications.

When applying LDPred, we tuned parameter *p* on the tuning data, and obtained prediction *r*^2^ from the testing data. The true value of *p* was contained in the set of tuning values for *p*, as were four other values; two larger than the true *p*, and two smaller. As per the recommendation of the original paper [10], we used *M*/3000 as the LD parameter, which controls the size of a sliding window of how many SNPs to consider when estimating joint effect sizes. When applying the polygenic risk score (denoted PRS), we used all marginal SNP effect size estimates from the training data. When applying the pruned and thresholded polygenic risk score (denoted PRS P+T), we first performed LD clumping in PLINK to ensure that no two SNPs were in LD *R*^2^ > .2. We then implemented a p-value cutoff, where only SNPs with marginal p-value below some cutoff were included in the risk score. The p-value cutoff was treated as a tuning parameter, and determined by maximizing accuracy on the tuning data. Both the LD *R*^2^ cutoff and the method of determining the p-value cutoff were done as in the LDPred paper [10]. The results are displayed in Figs 1, 2 and 3.

The penalized regression methods may have a slight advantage over LDPred when the ratio of SNPs to sample size N is smaller, as demonstrated by the simulation 1 results, while LDPred slightly outperforms the penalized methods slightly as N grows, as demonstrated by the simulation 2 results. In simulation 3, the penalized regression methods have roughly equivalent predictive accuracy to LDPred. In general, LDPred demonstrates similar predictive accuracy to the penalized regression methods across the three simulation settings. Additionally, it appears that the penalized regression methods perform comparatively better when *p* is smaller; that is, the signal is sparser. LDPred and the penalized regression methods outperform clumped polygenic risk scoring in all cases. The simple PRS performs poorly in most cases, except for when the fraction of causal SNPs *p* is large. These simulation results demonstrate that the penalized regression methods are competitive with LDPred in all simulation settings, and outperform PRS methods that do not account for linkage disequilibrium. In this simulation structure, we do not see much difference in performance between the three penalized regression methods.

#### Simulating allelic heterogeneity

The penalized regression methods LassoSum, TlpSum, and ElastSum demonstrate similar predictive performance in the previous section. Motivated by the concept of so-called ‘widespread allelic heterogeneity’ [27], we conduct a simulation where causal SNPs are clustered together in regions of high linkage disequilibrium. This simulates allelic heterogeneity, which is characterized by multiple SNPs within a single region (often a gene) that are causal for a trait. Under this simulation structure, we investigate the performance of the penalized regression methods, and demonstrate that TlpSum incurs modest but persistent gains in predictive accuracy as compared to LassoSum and ElastSum.

**Fig 4:**
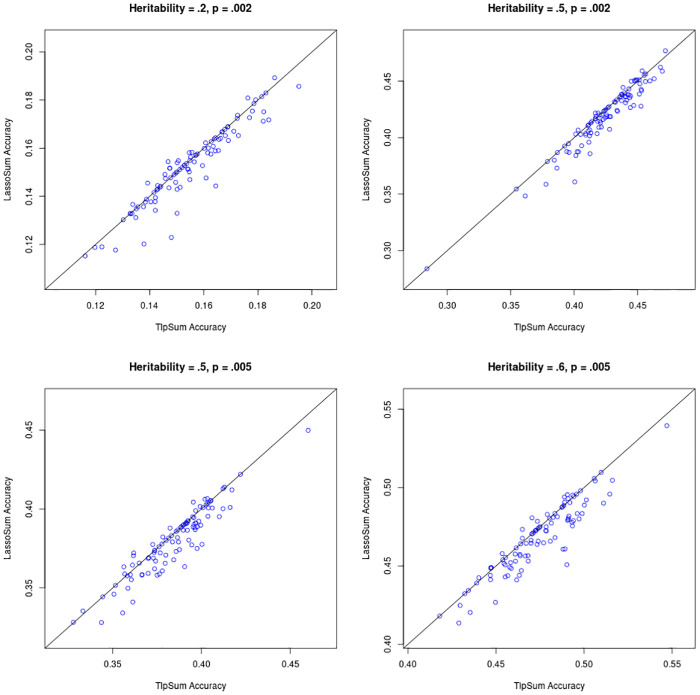
Predictive *r*^2^ on out-of-sample data for TlpSum and LassoSum for each of the 100 replications at each of the four simulation settings. Lines are at a 45 degree angle through the origin, and not a line of best fit. Points below the line indicate better performance of TlpSum.

**Fig 5:**
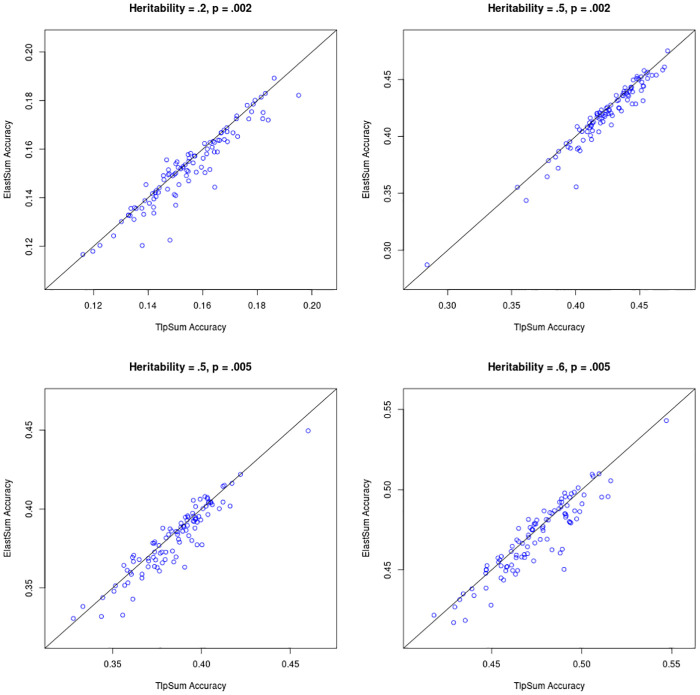
Predictive *r*^2^ on out-of-sample data for TlpSum and ElastSum for each of the 100 replications at each of the four simulation settings. Lines are at a 45 degree angle through the origin, and not a line of best fit. Points below the line indicate better performance of TlpSum.

We set up the simulation as follows. We use the ‘simulation 1’ structure from the previous section, with the following adjustments. Instead of simulating effect sizes from the point normal model with the probability of nonzero effect drawn independently for each SNP, we now simulate causal SNPs (i.e. SNPs with nonzero effect size) in groups of size 2 to 8. The process for simulating SNP effect sizes is described in **Section K in** S1 Text. We also adjusted the fraction of causal SNPs *p* and the SNP-based heritability *h*^2^. We considered values for *p* of.002 and.005, and values for *h*^2^ of .2, .5, .6. We conducted 100 replications at each simulation setting. In all four of the simulation settings considered, TlpSum had better predictive accuracy on out-of-sample data as compared to ElastSum and LassoSum. This improvement was measured to be statistically significant at *p* < .05 with a paired t-test. Figs 4 and 5 describe the performance of the TlpSum as compared to ElastSum and LassoSum across the four simulation settings. Additional results describing the relative performance of the LassoSum and the ElastSum, the results from some significance tests, and some results on predictive accuracy are located in **Section B in** S1 Text.

Figs 4 and 5 demonstrate the persistent advantage of the TlpSum as compared to the LassoSum and ElastSum when effect sizes are simulated under widespread allelic heterogeneity. This substantive improvement in predictive accuracy is evidence for the superior performance of the TlpSum as compared to other penalized regression methods for summary statistics in the context of widespread allelic heterogeneity.

#### Investigation of models fit by penalized regression

Many penalized regression methods impose a degree of sparsity on the estimated effect sizes. In particular, when the fraction of causal SNPs *p* is small, the proportion of nonzero estimated effects is generally also small. It is of interest to characterize this sparsity and examine how it might influence the predictive performance. In this section, we characterize the sparsity of the fitted penalized regression models. This issue bears some similarity to fine mapping, which includes methods such as CaviarBF [28] and FINEMAP [29]. We do not formulate formal hypothesis tests for variable selection in penalized regression in this paper, and we do not seek to compare our method to the fine mapping literature.

**Fig 6:**
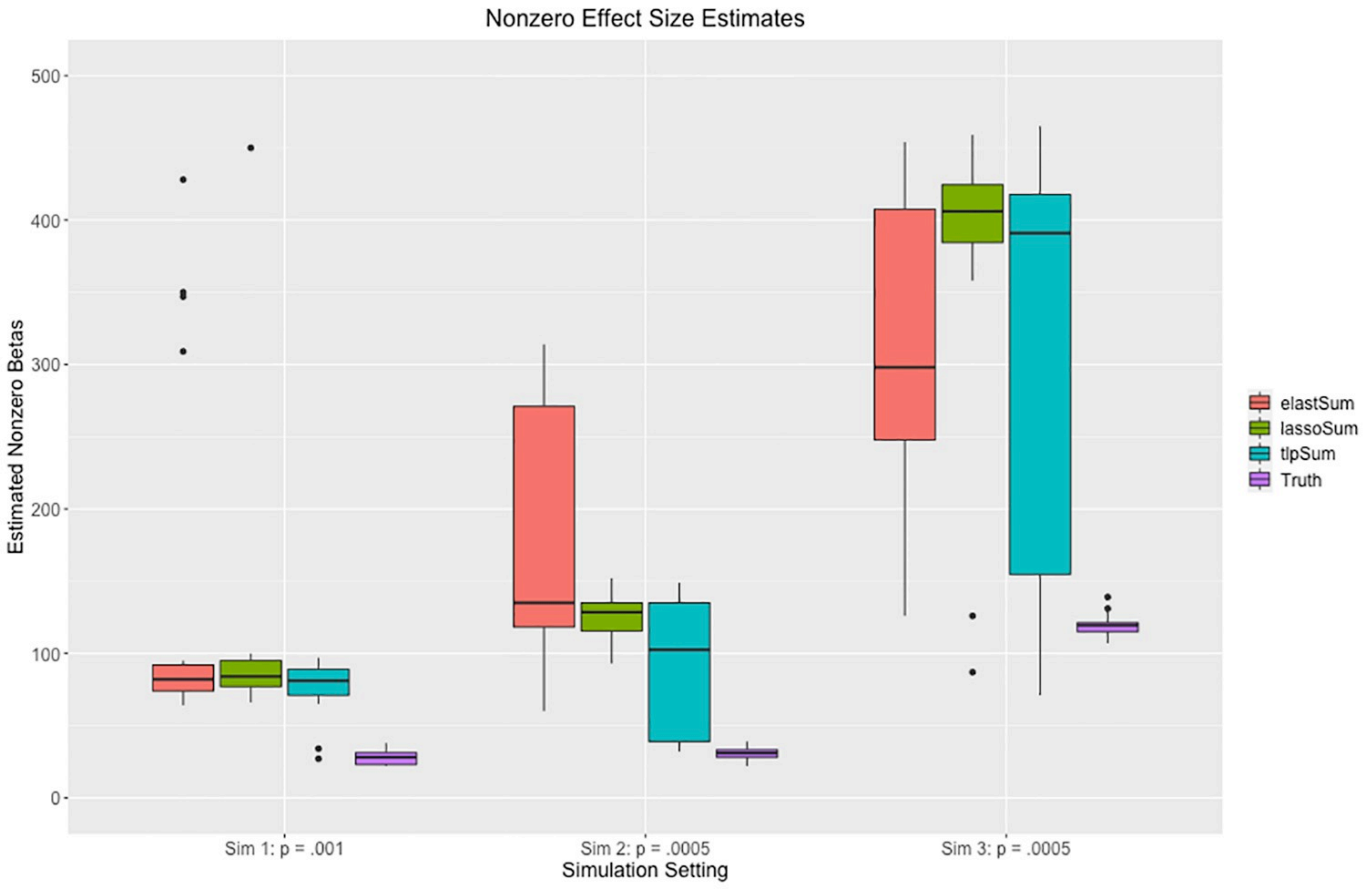
Number of nonzero effect sizes estimated by the three penalized regression methods as compared to the true number of nonzero effects, for the three sparse simulation settings.

Given that TLP does not penalize effect size estimates above a certain threshold, it may produce a smaller number of nonzero effect size estimates. This has been demonstrated in previous literature [16]. Thus, the TLP may be more parsimonious when the truth is sparse. We investigate the number of nonzero parameter estimates for sparse situations in simulations 1, 2, and 3. We find that the TLP produces sparser estimates than the LASSO and the elastic net in the case where *p* = .001 for simulation 1, *p* = .0005 for simulation 2, and *p* = .0005 for simulation 3. The results are not as clear for the cases where the fraction of causal SNPs *p* is larger. We suspect this is because all of the selected values for the tuning parameter *s* are nonzero in the case where *p* ∈ [.01, .1], and some of the time when *p* = .001 in simulations 2 and 3. This means that we do not have a “true” TLP, as is described in the methods section and illustrated in Eq (5). In the case where *p* = .001 in simulation 1 and *p* = .0005 in simulations 2 and 3, the optimal value of *s* is zero for all models or nearly all models, giving us a “true” TLP. We present the results for the three sparse simulation settings in Fig 6, while the full results are presented in **Section B in** S1 Text. Given that the models all achieve similar predictive performance on out of sample data as illustrated in Figs 1, 2 and 3, we see that the TLP can generate the same amount of predictive power with sparser models.

**Fig 7:**
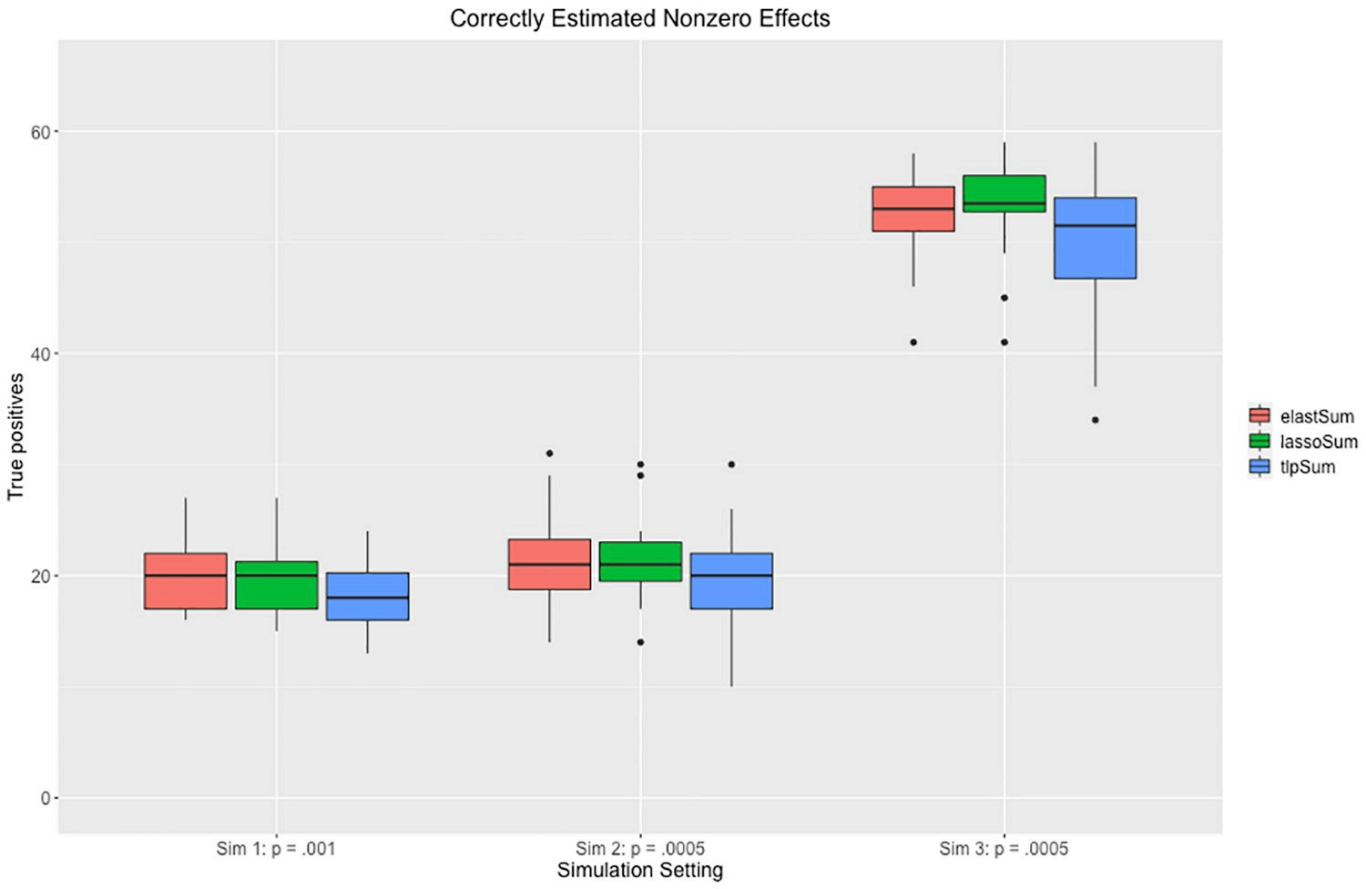
Number of true positives for the three penalized regression methods in the three sparse simulation settings.

**Fig 8:**
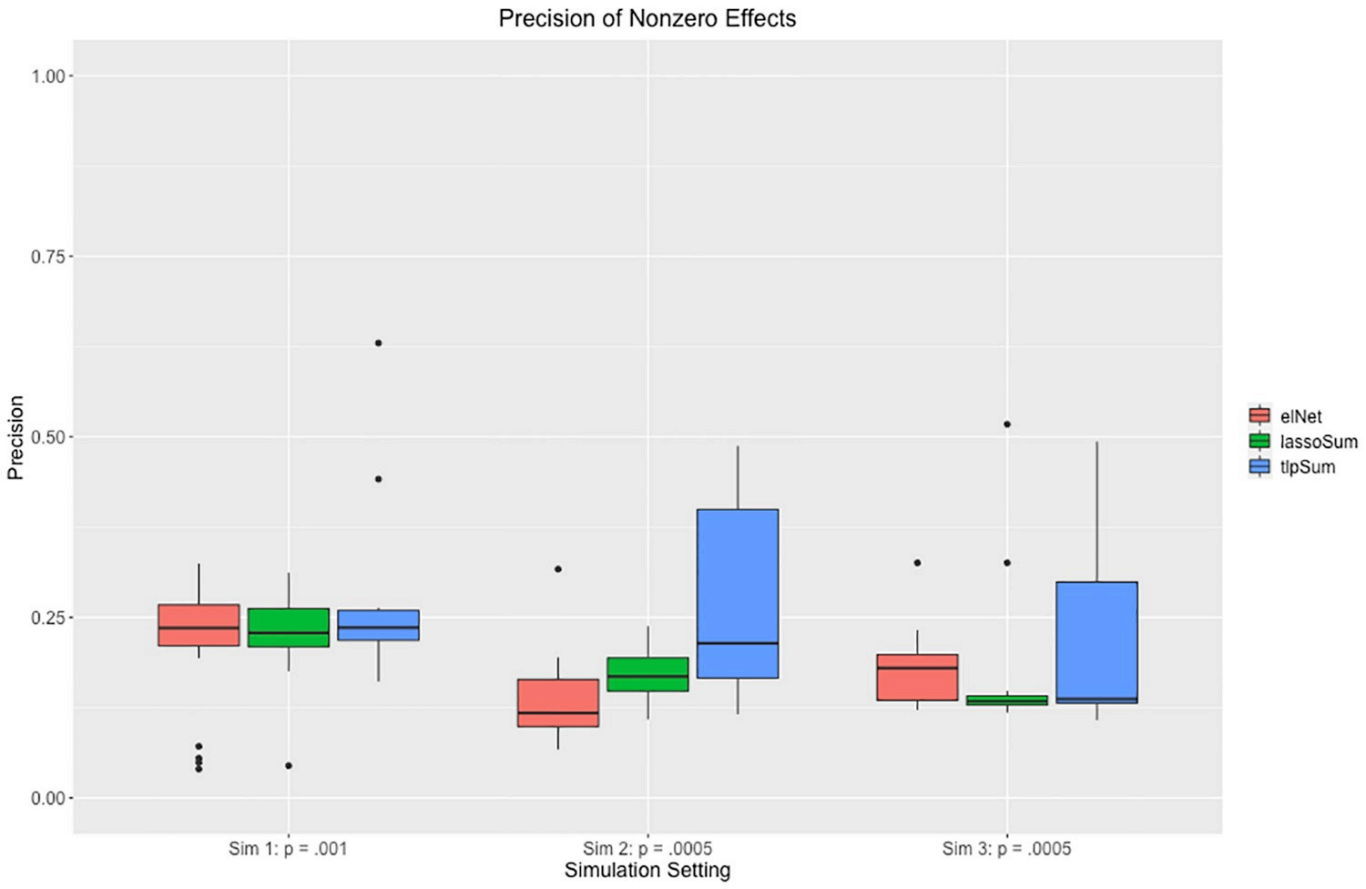
Precision of estimated nonzero effect sizes for the penalized regression methods applied to the three sparse simulation settings.

Also of interest is the number of true nonzero effects that are estimated to be nonzero by the penalized regression models. We can think of this as a binary prediction problem, where we are trying to predict which effects are nonzero. This information is presented in Fig 7 for the three sparse simulation settings. We see that the TLP has nearly the same number of true positives as the elastic net and LASSO, while having fewer total nonzero estimated effects, as displayed in Fig 6. This corresponds to a higher precision, as displayed in Fig 8. Note that precision corresponds to $\frac{TP}{TP+FP}$, where *TP* is the number of true positives, and *FP* is the number of false positives.

These simulation results provide evidence that, when the truth is sparse, the TLP may produce sparser effect size estimates and reduce the number of false positives, while capturing nearly the same number of true positives. This indicates that TLP models maintain predictive accuracy while being closest to the true structure of effects, thus facilitating the estimation of parsimonious models.

### Simulation study for model selection

Using the simulation structure without allelic heterogeneity as described previously, we assessed the comparative accuracy of model selection methods. We compared the three model selection methods we proposed, namely the pseudo AIC, pseudo BIC, and quasi-correlation, to existing model selection method pseudovalidation. Note that these four model selection methods do not require the existence of individual level tuning or training data, and are thus more widely applicable, especially in the framework of summary statistics and reference panels. As a point of comparison, we also include the performance of AIC and BIC for the model as fit on the training data (the so-called ‘true AIC’ and ‘true BIC’). The true AIC and true BIC assume that we have access to individual level genotype data for the training dataset, which is not generally the case. They also directly use the true residual variance ${\tilde{\sigma }}^{2}$, which must be estimated in practice. We also compare the performance of selecting the model with maximum *r*^2^ on the tuning data, which is a widely applied model selection criteria. This assumes that we have individual level phenotype data for the tuning dataset, which may not be the case.

We split the WTCCC data into four disjoint datasets as described below. This allowed us to simulate a setting where our proposed pseudo AIC / BIC and quasi-correlation could be estimated in a realistic setting. As described in the methods section, it is important not to reuse the same reference panel for the penalized regression methods and the model fitting methods. This explains the presence of two ‘tuning’ datasets. This practice, where we essentially split the reference panel in half and use one half for the penalized regression methods and the other half for the model fitting methods, is used in our real data applications as well. The four datasets are as follows:

- The training data **X**_*tr*_, which we used to estimate univariate summary statistics for each SNP. **X**_*tr*_ had sample size 6240.
- The tuning-1 data **X**_*tu*1_, which was used as a refernece panel for the model estimation methods, namely TlpSum and LassoSum. **X**_*tu*1_ had sample size 3119.
- The tuning-2 data **X**_*tu*2_, which was used as a reference panel for the model selection metrics that required a reference panel: namely pseudo AIC, pseudo BIC, pseudovalidation, and quasi-correlation. **X**_*tu*2_ had sample size 1560.
- The testing data **X**_*te*_, which was used to evaluate the performance of the polygenic risk scores. **X**_*te*_ had sample size 1560.

For this simulation study, we used simulation setting 1: that, is, we used SNPs from chromosomes 1 and 4 from the WTCCC study, and simulated phenotypes from the point-normal model, varying the fraction of causal SNPs *p*. We used the same filtering steps as described previously. We estimated univariate summary statistics from the training data (*N* = 6240). We then used the tuning-1 data (*N* = 3119) as a reference panel to estimate polygenic risk scores using TlpSum and LassoSum. For TlpSum, we used a three dimensional matrix of tuning parameters λ, *s*, *τ* to generate a set of candidate polygenic risk scores. For LassoSum, we used a two dimensional matrix of tuning parameters λ, *s* to generate a set of candidate polygenic risk scores. The results from applying the model selection metrics to LassoSum are presented here. The results of applying the model selection metrics to the TlpSum models, which are similar, are located in **Section B in** S1 Text.

For the estimation of the pseudo AIC, pseudo BIC, quasi-correlation, and pseudovalidation, we used the tuning-2 dataset (*N* = 1560) as a reference panel. Although pseudovaldiation does not require the tuning data to be split in half as pseudo AIC and pseudo BIC do, we note that using the split tuning data versus the full tuning data made no difference in practice for pseudovalidation. For the quasi-correlation criteria we used summary statistics estimated from the tuning-2 data. Using the seven model fitting criteria that we described, we selected a best model in accordance with each of the criteria. We then measured the predictive *r*^2^ of that model applied to the testing data. This was repeated for each of the 20 simulations, across three different values of *p*, the fraction of causal SNPs.

**Fig 9:**
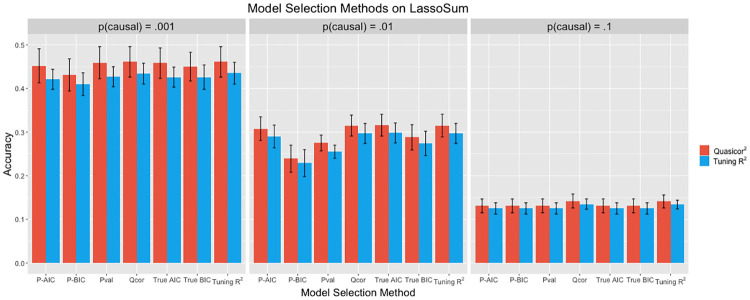
Performance of the seven different model selection methods applied to a set of candidate LassoSum models. Performance is measured by *r*^2^ on the testing data (the right bar in each group), and by squared quasi-correlation on the testing data (the left bar in each group). Error bars represent the standard deviation across 20 replications.

In addition to considering quasi-correlation as a model selection metric, we have also proposed using quasi-correlation as a measure of model fit; that is, as a way to compare the performance of different models. In the case where we do not have individual level testing data, we will not be able to use many common measures of predictive performance. If we have access to summary statistics from the testing data, we will be able to use quasi-correlation. We want the relative performance of the different model selection methods as measured by predictive *r*^2^ on the testing data to be the same as the relative performance as measured by quasi-correlation. Note that we have two different applications of quasi-correlation here; we are using it for model selection, and to quantify model performance. Quasi-correlation for model selection is estimated using summary statistics from the tuning-2 data; this corresponds to the ‘Qcor’ bar group in the bar chart. Quasi-correlation for quantifying model performance is estimated using summary statistics from the testing data; this corresponds to the red bars in the bar chart. The results are displayed in Fig 9.

These results show that quasi-correlation performs well as a model selection method, outperforming all other metrics except for tuning *r*^2^, which it performs equivalently to. The pseudo AIC and pseudo BIC perform relatively similarly to the true AIC and true BIC, although the true AIC and BIC do perform equivalently or better in all cases, which is to be expected. Additionally, we see that the pseudo AIC outperforms pseudovalidation in the case where *p* = .01. The methods perform similarly when *p* = .001 and *p* = .1. In this simulation, pseudovalidation, pseudo AIC, and pseudo BIC all appear to be reasonable methods for model selection when validation data is not available. A more thorough analysis of the accuracy of the different components of the estimation of pseudo AIC / BIC and quasi-correlation is located in **Section D in** S1 Text.

We also assess the usefulness of quasi-correlation as a measure of model fit. Fig 9 shows that the relative performance of the model selection methods as measured by quasi-correlation squared and testing *r*^2^ are generally equivalent. Quasi-correlation appears to slightly overestimate the testing *r*^2^ a majority of the time, and the standard deviation across the twenty replications is a somewhat larger. Nevertheless, we can conclude that quasi-correlation does a good job approximating the testing *r*^2^ given the high degree of similarity between the testing *r*^2^ and squared quasi-correlation estimates.

These results demonstrate that quasi-correlation approximates the predictive performance of selected models well on average. Also of interest is how well quasi-correlation performs within a single replication, i.e. whether quasi-correlation can generally differentiate between the predictive performance on out-of-sample data for a set of candidate models. This particularly concerns the performance of a set candidate models on a single out-of-sample dataset, rather than the average across twenty replications as shown in Fig 9. Results described in **Section D in** S1 Text indicate that the quasi-correlation generally does this well.

#### Investigation of selected models

We examine the model selection performance of pseudo AIC and pseudo BIC as applied to penalized regression models in the summary statistic framework, and generally conclude that they demonstrate good performance.

**Fig 10:**
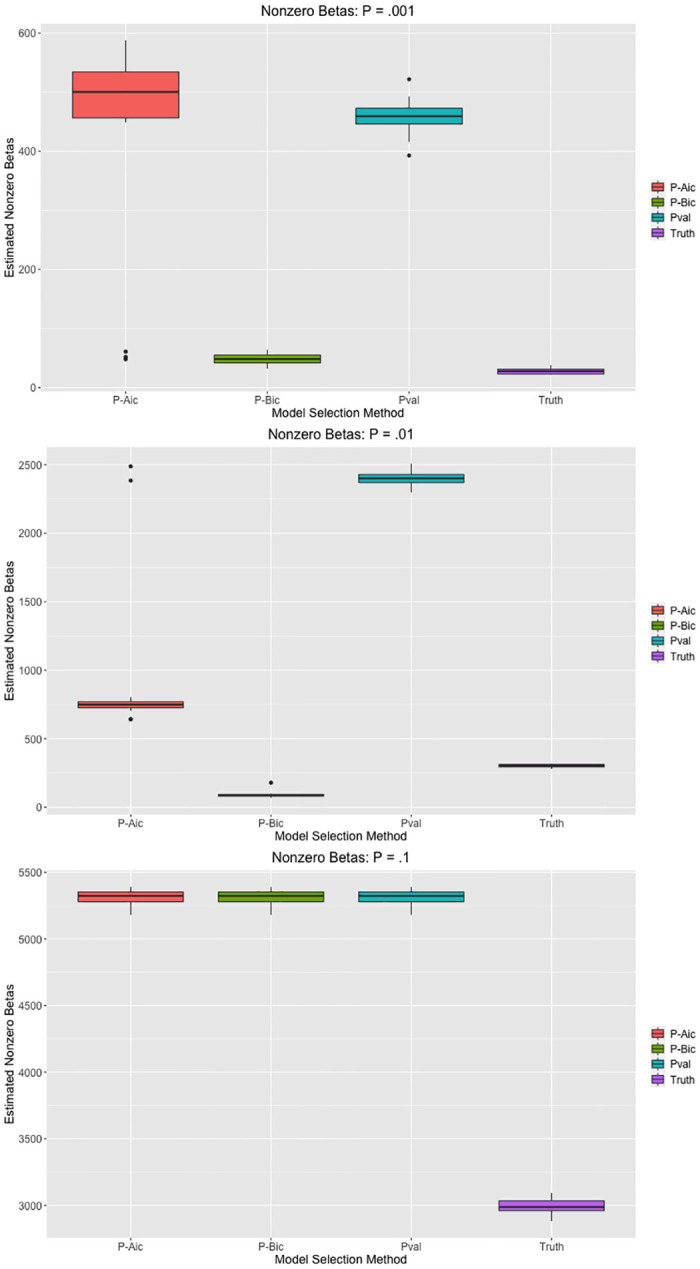
Number of estimated nonzero effects for each model selection method across each of the simulation settings in simulation 1. Models were selected from a set of candidate LassoSum models.

**Fig 11:**
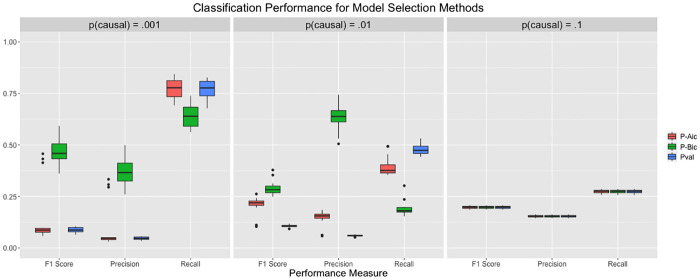
Performance of the selected models for each of the model selection methods across the different simulation settings of simulation 1, as measured by precision, recall, and F1 score. The leftmost box in each grouping of three corresponds to pseudo AIC, the center corresponds to pseudo BIC, and the rightmost corresponds to pseudovalidation. Models were selected from a set of candidate LassoSum models.

Via simulation, we show that pseudo AIC and pseudo BIC select sparser models than pseudovalidation, and that pseudo AIC and pseudo BIC generally reproduce the true model more accurately. In particular, we consider simulation setting 1 without allelic heterogeneity as described previously. For each of the 20 replications at each of the three fractions of causal SNPs *p*, we compare the number of nonzero effect sizes for each of the three model selection methods to demonstrate the tendency of the methods to select models of differing sparsity. We describe the precision, recall, and F1 score for the model selected by each the three model selection methods to demonstrate the degree to which selected models recapture the true model. These measures of accuracy are considered in the following context. A ‘true positive’ occurs when a SNP with a nonzero effect size has an estimated nonzero effect in the corresponding model. Likewise, a false positive occurs when a model estimates a SNP effect to be nonzero and the true SNP effect is zero. If we define *TP* as the number of true positives captured by a model, *FP* as the number of false positives, and *FN* as the number of false negatives, we can define precision as $\frac{TP}{TP+FP}$ and recall as $\frac{TP}{TP+FN}$. The F1 score is defined as $F_{1}=2\frac{precision\times recall}{precision+recall}$, which is the harmonic mean of precision and recall. We consider the application of model selection to a set of candidate LassoSum models; we believe that the performance would be similar for TlpSum. We expect that the pseudo AIC and pseudo BIC may select sparser models than pseudovalidation, and that the selected models may perform better as measured by precision and F1 score. We generally expect the models selected by pseudovalidation to display better recall, given that they have more estimated nonzero effects. The results are displayed in Figs 10 and 11.

We see that the three model selection methods perform equivalently when *p* = .1, but there is discrepancy when *p* < .1. Pseudo BIC selects substantially sparser models than either pseudo AIC or pseudovalidation, while pseudo AIC selects somewhat sparser models than pseudovalidation. Fig 11 shows that pseudo BIC substantially outperforms pseudo AIC and pseudovalidation according to the precision and F1 score metrics, although pseudo BIC performs less well as measured by recall. Pseudo AIC outperforms pseudovalidation as measured by precision and F1 score as well. Given that F1 score can be considered an overall measure of binary classification performance that considers precision and recall, it is reasonable to state that pseudo BIC substantially outperforms the other two model selection methods, indicating that it best reproduces the true model.

Pseudo AIC and pseudo BIC impose model sparsity according to established theory, while pseudovalidation selects a model by minimizing training error under an ad hoc condition that imposes some sparsity. The previous section demonstrates that these three selection methods perform reasonably similarly as measured by predictive accuracy on out-of-sample data, but there is moderate discrepancy among the three methods in ability to reproduce the true model. In applications where it is important to select only those variants that are truly associated, such as the selection of valid instruments for a TWAS-type analysis [30], it may be preferable to use pseudo AIC or pseudo BIC.

### Application to lipids

We leverage our methodology to perform model estimation and model selection for GWAS analyses of lipid data. We estimate models based on summary statistics from the Teslovich et al. study [31]. We assess model accuracy via quasi-correlation, using summary statistics from the UK BioBank as our out-of-sample data [32]. For partial validation and the estimation of quasi-correlation for model selection on a third dataset, we use summary statistics from the Global Lipids Genetics Consortium, or GLGC [33]. We consider three different phenotypes in this analysis: high-density lipoprotien (HDL), low-density lipoprotein (LDL), and triglycerides (TG). We use the 1000G data as a reference panel [34], and limit the reference panel to only those individuals of European ancestry.

For each study, we did quality control as follows. We removed SNPs with *MAF* < .01 in the reference panel or in the Teslovich data. We then determined the subset of SNPs that was present in all four datasets: the Teslovich data, the GLGC data, the BioBank data, and the 1000G data. We excluded all SNPs not in the intersection of these datasets. We did LD clumping using the 1000G data as a reference panel and univariate p-values from the Teslovich data, ensuring that no two SNPs were in LD *R*^2^ > .9. This was done to ensure convergence of our penalized regression methods, and shouldn’t substantively affect the results, given that we don’t expect many informative SNPs to be pruned away. We removed all ambiguous SNPs (i.e. those SNPs with alleles A/T or C/G), and all SNPs with allele coding irreconcilably different between the datasets. After quality control, we had 640,675 SNPs for TG, 639,754 SNPs for LDL, and 642,675 SNPs for HDL. The Teslovich and GLGC studies are meta-analyses, so the sample size varies by SNP. The BioBank study has equal sample size for all SNPs. We present the median sample size for each study and phenotype in Table 1.

**Table 1:** Median sample size for each study in the lipid analysis.

	Teslovich	GLGC	BioBank
TG	95,877	90,976	343,992
LDL	94,769	89,855	343,621
HDL	99,179	94,277	315,133

Using these sets of SNPs, we estimated a set of 48 candidate polygenic risk scores for each lipid phenotype. We estimated polygenic risk scores via TlpSum using 48 unique sets of tuning parameters *τ*, *s* and λ, with the summary statistics from the Teslovich study as our training data. Application of model selection methods to LassoSum models produced similar results, as demonstrated in **Section J in** S1 Text. We did not apply model selection methods to LDPred models. As LDPred models impose no sparsity, the penalty on model size imposed by pseudo AIC and pseudo BIC cannot be interpreted in a meaningful way. Thus, these selection methods degenerate into simply using estimated training SSE as a criteria for model selection, which is not particularly useful. This is an advantage of penalized regression methods that impose sparsity as compared to LDPred; namely, that models that impose sparsity can leverage the pseudo AIC and pseudo BIC for model selection.

We split the 1000G data into two groups of equal sample size as described in the methods section, and used one half of this data as the reference panel for estimating the TlpSum models. The other half was used to estimate the model fitting criteria. We then estimated model fitting criteria pseudo AIC, pseudo BIC, and pseudovalidation. We also estimated quasi-correlation for model selection by using the GLGC study as our out-of-sample data. There is substantial overlap between the samples used in the GLGC study and the Teslovich study; however, given that the study populations are not identical, we believe it is reasonable to apply the quasi-correlation here.

We present the accuracy of each method, as measured by quasi-correlation on the BioBank data, in Table 2. In this case, none of the model selection methods perform particularly well, given that all methods select a model that performs worse than the best performing model. The accuracy of the best performing model is quantified in the ‘Maximum’ column, and represents the maximum quasi-correlation attained by any of the 48 candidate models predicted into the BioBank data. We see that the pseudo AIC, pseudo BIC, and quasi-correlation all outperform pseudovalidation for all three lipid phenotypes. Given the relatively small amount of heritability captured, even by the best performing models, and the smaller sample size of the Teslovich study, this is likely a scenario where it is important to impose model sparsity during model selection. Because the pseudo AIC and pseudo BIC impose more model sparsity than pseudovalidation, and tend to be more parsimonious in recapturing the true model, the performance of the models selected by pseudo AIC and pseudo BIC are superior to the model selected by pseudovalidation in this application. On balance, the best performing model selection method is quasi-correlation, given that it selects the model with the best performance for two of the three lipid phenotypes. This is reasonable, given that quasi-correlation for model selection leverages information from a third dataset.

**Table 2:** Model performance, as measured by quasi-correlation of the model predicted into the BioBank data, for each model selection method. Models were estimated via TlpSum on the Teslovich data.

	Quasi-cor	Pseudo AIC	Pseudo BIC	Pseudoval	Maximum
TG	.14	.13	.11	.10	.22
LDL	.12	.16	.14	.11	.21
HDL	.20	.18	.18	.17	.30

In this application, we demonstrate that pseudo AIC and pseudo BIC select models with superior predictive accuracy on out-of-sample data as compared to pseudovalidation for all three lipid phenotypes. We demonstrate the usefulness of quasi-corrleation for model selection given a third dataset by showing that it selects models with good predictive accuracy on out-of-sample data. Likewise, we use quasi-correlation to assess predictive performance on out-of-sample data. Without quasi-correlation, it would not be possible to leverage summary statistic data as out-of-sample data for this purpose.

## Discussion

In this paper, we propose applying the Truncated Lasso penalty and the elastic net penalty to calculate polygenic risk scores using summary statistic data and linkage disequilibrium information. We demonstrate via simulation that the TlpSum produces sparser models when the underlying genetic architecture is sparse, and does a good job recovering truly nonzero effect sizes while limiting false positives. Additionally, we demonstrate that the TlpSum improves predictive accuracy as compared to other penalized regression models when applied to data simulated under widespread allelic heterogeneity. We propose methods for estimating model fit statistics AIC and BIC for polygenic risk scores in the case where we have only summary statistic data and linkage disequilibrium information. This facilitates model selection in the case where we do not have access to validation data. This complements existing method pseudovalidation, which may tend to select overfit models. We also propose the so-called quasi-correlation, which allows us to quantify the predictive accuracy of a polygenic risk score on out-of-sample data for which we have only summary statistic information. Quasi-correlation can also be used to leverage information from a third ‘tuning’ dataset of summary statistics for model selection. These methods in totality broaden the scope of the application of polygenic risk scores. Using only summary statistics and publicly available reference panels, we can estimate polygenic risk scores, perform model selection given a candidate set of polygenic risk scores, and quantify the predictive accuracy of these polygenic risk scores on out-of-sample summary statistic data. This facilitates the construction of validated polygenic risk scores ready for use on new data. Additionally, it facilitates the application of polygenic risk scores to large summary statistic data, generating robust models based on large studies. These models can be used infer the genetic architecture of complex phenotypes.

We demonstrate via simulation that penalized regression with the TLP penalty performs well as compared to existing methods, improving predictive performance in the context of allelic heterogeneity and inducing sparsity when the true model is sparse. We investigate the comparative performance of the pseudo AIC, pseudo BIC, pseudovalidation, and quasi-correlation for model selection via simulation, demonstrating that quasi-correlation performs well in all cases, and that pseudo AIC and pseudo BIC outperform pseudovalidation in some cases. Pseudo AIC and pseudo BIC demonstrate some desirable model selection properties in simulation, with pseudo BIC in particular tending to recover the true model better than pseudovalidation. We also show via simulation that quasi-correlation approximates the actual predictive *r*^2^ well, indicating that it is an appropriate and robust measure of model fit. We demonstrate the usefulness of pseudo AIC and BIC and quasi-correlation for model selection by demonstrating their superior performance to pseudovalidation in an application to a large GWAS of lipid data. In **Section F in** S1 Text, we apply penalized regression and model fitting methods to a large lung cancer meta-analysis, demonstrating that penalized regression methods improve accuracy as compared to simple polygenic risk score methods. We additionally demonstrate the application of pseudo AIC and BIC methods to a GWAS analysis with a binary phenotype. In **Section G in** S1 Text, we apply penalized regression and model fit methods to large summary statistic data of the height phenotype, which allows us to assess the performance of our penalized regression methodology and model selection methods on a large GWAS for a highly heritable phenotype.

## Supporting information

S1 Text Supplementary file with additional simulation results, additional real data results, descriptions of some algorithms, mathematical discussion of assumptions and derivations. (PDF)
