# Supplementary material for: Penalized regression and model selection methods for polygenic scores on summary statistics
Source: PLoS Comput Biol. 2020 Oct 1;16(10):e1008271. doi: 10.1371/journal.pcbi.1008271 (PMC7553329; doi:10.1371/journal.pcbi.1008271)
Supplement: S1 Text — (PDF) [file pcbi.1008271.s001.pdf]

Supplementary Materials for "Penalized Regression and Model Selection Methods for Polygenic Scores on Summary Statistics"

Jack Pattee[1] and Wei Pan[2]

[1]Current address: Division of Biostatistics, School of Public Health, University of Minnesota, Minneapolis, Minnesota 55455. Email: patte631@umn.edu. Phone: 612-716-7470. Fax: 612-626-0660.
[2]Current address: Division of Biostatistics, School of Public Health, University of Minnesota, Minneapolis, Minnesota 55455. Email: panxx014@umn.edu. Phone: 612-626-2705. Fax: 612-626-0660.

March 2020; revised June 2020

A Coordinate Descent Algorithms

We provide a software to perform the estimation of the penalized regression models described above. The software solves the objective function (5) via coordinate descent.

First, we describe the coordinate descent algorithm for the LASSO penalty. We have the penalized regression objective function, standardized $n \times p$ design matrix $\mathbf{X}$, and standardized response vector $\mathbf{y}$. Let our vector of temporary effect size estimates, which is updated elementwise by the coordinate descent algorithm, be denoted $\tilde{\boldsymbol{\beta}}$. We loop over $\tilde{\boldsymbol{\beta}}$ to update each element $\tilde{\beta}_j$ in sequence. We iterate this process until convergence, which is defined as two successive iterations that produce no elementwise change above some (small) threshold. The updating formula is as follows [1]:

$$\tilde{\beta}_j \leftarrow S(\sum_{i=1}^{n} x_{ij}(y_i - \tilde{y}_i^{(j)}), \lambda) \tag{S.1}$$

where S is the soft-thresholding operator, and $\tilde{y}_i^{(j)} = \sum_{k \neq j} x_{ik}\tilde{\beta}_k$. We can also express the updating formula as:

$$\tilde{\beta}_j \leftarrow S(\sum_{i=1}^{n} x_{ij}y_i - x_{ij}\sum_{k \neq j} x_{ik}\tilde{\beta}_k, \lambda) \tag{S.2}$$

We can make substitutions equivalent to equation (4) to derive an updating formula that can be used in our framework of summary statistics and reference data. We also define the following quantity: $\tilde{\boldsymbol{\beta}}_{j=0}$ is equal to $\tilde{\boldsymbol{\beta}}$ with the jth element equal to zero. Given these, we can represent equation (S.2) as:

$$\tilde{\beta}_j(\lambda) \leftarrow S([\mathbf{r} - \mathbf{R_s}\tilde{\boldsymbol{\beta}}_{j=0}(\lambda)]_j, \lambda) \tag{S.3}$$

where $[\mathbf{r} - \mathbf{R}\tilde{\boldsymbol{\beta}}_{j=0}(\lambda)]_j$ denotes the jth element of the vector. The above coordinate descent algorithm for summary statistic data is implemented by Shin et al [2] in their LassoSum package.

A similar process follows for the TLP and the elastic net. In the elastic net, we have the following updating formula [1]:

$$\tilde{\beta}_j = \frac{S(\sum_{i=1}^{n} x_{ij}(y_i - \tilde{y}_i^{(j)}), \lambda\alpha)}{1 + (1-\alpha)\lambda} \tag{S.4}$$

We make the substitutions specified above to get the following update formula which allows us to estimate the model in the summary statistic framework:

$$\tilde{\beta}_j = \frac{S([\mathbf{r} - \mathbf{R_s}\tilde{\boldsymbol{\beta}}_{j=0}]_j, \lambda\alpha)}{1 + (1-\alpha)\lambda} \tag{S.5}$$

We now present the updating formula for the TLP [3]. We introduce the following notation: consider that estimated effect size $\tilde{\beta}_j^{(m)}$ is from the mth iteration of the coordinate descent algorithm. Given this, we define the updating formula as follows:

$$\tilde{\beta}_j^{(m)} \leftarrow \begin{cases} \sum_{i=1}^n x_{ij}(y_i - \tilde{y}_i^{(j)}), & \text{if } \tilde{\beta}_j^{(m-1)} > \tau \\ S(\sum_{i=1}^n x_{ij}(y_i - \tilde{y}_i^{(j)}), \lambda), & \text{if } \tilde{\beta}_j^{(m-1)} < \tau \end{cases} \tag{S.6}$$

Which gives us the following update formula after substituting:

$$\tilde{\beta}_j^{(m)}(\lambda) \leftarrow \begin{cases} [\mathbf{r} - \mathbf{R_s}\tilde{\beta}_{j=0}(\lambda)]_j, & \text{if } \tilde{\beta}_j^{(m-1)} > \tau \\ S([\mathbf{r} - \mathbf{R_s}\tilde{\beta}_{j=0}(\lambda)]_j, \lambda), & \text{if } \tilde{\beta}_j^{(m-1)} < \tau \end{cases} \tag{S.7}$$

B Additional Results from Simulation Study

Here, we present the information plotted in Fig 1, 2, and 3 in table form.

P	LDPred	LDP-Inf	LassoSum	TlpSum	ElNet	PRS	PRS P+T
.001	.411 (.030)	.125 (.020)	.437 (.029)	.440 (.029)	.436 (.028)	.123 (.022)	.380 (.024)
.01	.264 (.023)	.123 (.011)	.300 (.017)	.300 (.017)	.300 (.017)	.122 (.014)	.251 (.018)
.1	.132 (.012)	.123 (.011)	.134 (.013)	.136 (.013)	.135 (.013)	.123 (.012)	.123 (.012)

Table A: Prediction r^2 values for simulation 1. Standard deviation across the 20 replications in parentheses. The penalized regression methods have an advantage over LDPred as p decreases.

P	LDPred	LDP-Inf	LassoSum	TlpSum	ElNet	PRS	PRS P+T
.0005	.437 (.021)	.072 (.023)	.423 (.028)	.426 (.025)	.423 (.027)	.071 (.022)	.358 (.018)
.001	.412 (.025)	.078 (.016)	.397 (.029)	.399 (.027)	.398 (.028)	.075 (.017)	.346 (.032)
.01	.219 (.015)	.076 (.009)	.202 (.016)	.202 (.016)	.205 (.016)	.074 (.009)	.161 (.103)
.1	.076 (.010)	.072 (.009)	.075 (.009)	.074 (.009)	.075 (.009)	.072 (.010)	.072 (.010)

Table B: Prediction r^2 values for simulation 2. Standard deviation across the 20 replications in parentheses. LDPred is the most accurate in all scenarios.

P	LDPred	LDP-Inf	LassoSum	TlpSum	ElNet	PRS	PRS P+T
.0005	.347 (.068)	.033 (.008)	.348 (.048)	.353 (.043)	.349 (.049)	.024 (.006)	.302 (.034)
.001	.265 (.082)	.028 (.008)	.274 (.051)	.277 (.045)	.274 (.051)	.025 (.007)	.234 (.038)
.01	.037(.011)	.024 (.004)	.032 (.011)	.033 (.011)	.032 (.011)	.023 (.006)	.028 (.010)

Table C: Prediction r^2 values for simulation 3. Standard deviation across the 20 replications in parentheses.

Following are tables corresponding to the section on simulating allelic heterogeneity. This includes information on the average accuracy of the penalized regression methods applied to out-of-sample data simulated under allelic heterogeneity, paired t-tests that demonstrate a small but persistent improvement in predictive accuracy by the TlpSum, and a comparison of the predictive performance of LassoSum and ElastSum.

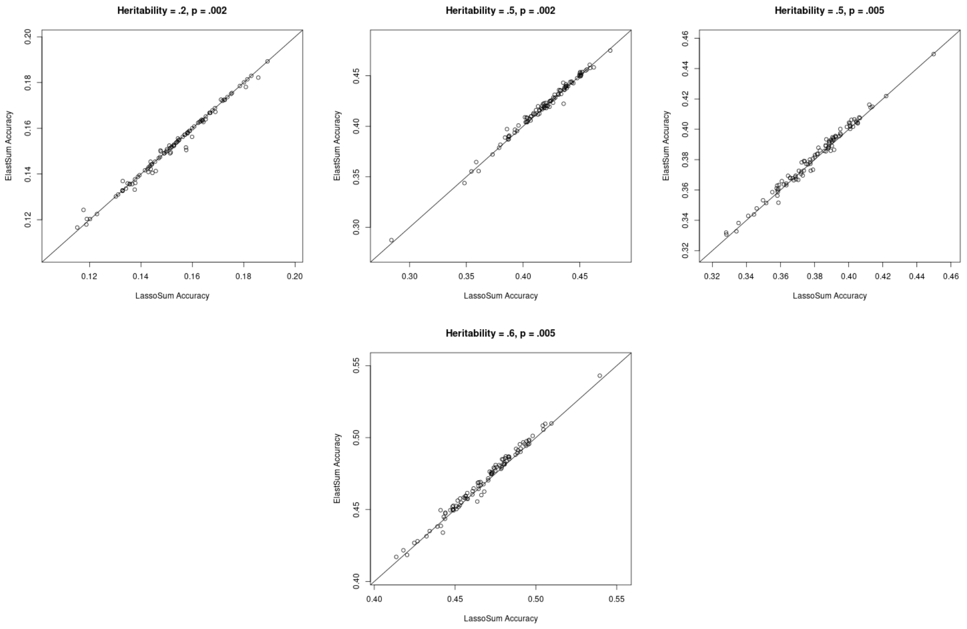

Fig A: Predictive r^2 for LassoSum and ElastSum for each of the 100 replications at each of the four simulation settings. Lines are at a 45 degree angle through the origin, and not a line of best fit. Points below the line indicate better performance of LassoSum.

	ElastSum	TlpSum
LassoSum	2.37E-10 (.001, .002)	3.05E-10 (.004, .008)
ElastSum		1.17E-5 (.002, .006)

Table D: P-values and 95% CIs for paired t-test applied to predictive r^2 on out of sample data for the allelic heterogeneity simulation. Ranges are for the method in the column label less the method in the row label. Simulation setting with $h^2 = .6, p = .005$.

	ElastSum	TlpSum
LassoSum	1.18E-6 (.001, .002)	2.69E-8 (.003, .006)
ElastSum		4.32E-5 (.002, .005)

Table E: P-values and 95% CIs for paired t-test applied to predictive r^2 on out of sample data for the allelic heterogeneity simulation. Ranges are for the method in the column label less the method in the row label. Simulation setting with $h^2 = .5, p = .005$.

	ElastSum	TlpSum
LassoSum	2.30E-5 (.001, .002)	1.61E-10 (.004, .007)
ElastSum		5.57E-8 (.003, .006)

Table F: P-values and 95% CIs for paired t-test applied to predictive r^2 on out of sample data for the allelic heterogeneity simulation. Ranges are for the method in the column label less the method in the row label. Simulation setting with $h^2 = .5, p = .002$.

	ElastSum	TlpSum
LassoSum	.075 (-6.37E-4, 3.16E-5)	6.59E-5 (.001, .003)
ElastSum		5.98E-6 (.001, .004)

Table G: P-values and 95% CIs for paired t-test applied to predictive r^2 on out of sample data for the allelic heterogeneity simulation. Ranges are for the method in the column label less the method in the row label. Simulation setting with $h^2 = .2, p = .002$.

Simulation	LassoSum	TlpSum	ElastSum
$h^2 = .6, p = .005$	.468 (.022)	.474 (.022)	.470 (.023)
$h^2 = .5, p = .005$	.380 (.021)	.385 (.020)	.381 (.021)
$h^2 = .5, p = .002$	.419 (.029)	.424 (.027)	.420 (.028)
$h^2 = .2, p = .002$	.153 (.056)	.155 (.056)	.152 (.056)

Table H: Predictive r^2 on out-of-sample data for TlpSum and LassoSum models in four simulations for allelic heterogeneity. Number in parentheses is standard deviation across 100 replications.

Following are tables corresponding to the section on the investigation of selected models, including information from those 'non-sparse' simulation settings as a point of comparison. In these tables, we clearly see that the increased sparsity and decreased false positive rate demonstrated by the TLP in the sparse simulation settings does not extend to those non-sparse simulation settings. Again, this is likely because we do not have a 'true' TLP when the s parameter is nonzero, as demonstrated by equation (5).

P	True	LassoSum	TlpSum	ElNet
.001	27.7	102.7 (82.3)	76.4 (18.9)	135.8 (116.2)
.01	302.4	631.3 (46.7)	631.2 (48.5)	597.2 (55.2)
.1	2995.7	14,107 (3378)	14,646 (3424.9)	13,640 (2,888)

Table I: Comparing the mean number of nonzero effect size estimates of the penalized regression methods against the ground truth in simulation 1. The standard deviation across the 20 simulations is in parentheses. For the penalized regression estimates, the number of nonzero effect size estimates corresponds to the model with the best tuning accuracy.

P	True	LassoSum	TlpSum	ElNet
.0005	30.6	124.75 (15.5)	90 (46.9)	187.7 (85.6)
.001	61.8	150.3 (13.1)	207.2 (236.1)	259.5 (80.6)
.01	617.4	1170.0 (233.2)	1222.6 (122.6)	809 (85.4)
.1	6128.8	36,223 (10,434)	38,125 (9953)	43,069 (5662)

Table J: Comparing the mean number of nonzero effect size estimates of the penalized regression methods against the ground truth in simulation 2. The standard deviation across the 20 simulations is in parentheses. For the penalized regression estimates, the number of nonzero effect size estimates corresponds to the model with the best tuning accuracy.

P	True	LassoSum	TlpSum	ElNet
.0005	119.6	381.6 (98.1)	322.1 (141.9)	321.6 (94.2)
.001	233.1	433.9 (81.7)	391.6 (119.6)	383.2 (92.7)
.01	2312	4185 (5777)	4872 (6643)	7317 (16,090)

Table K: Comparing the mean number of nonzero effect size estimates of the penalized regression methods against the ground truth in simulation 3. The standard deviation across the 20 simulations is in parentheses. For the penalized regression estimates, the number of nonzero effect size estimates corresponds to the model with the best tuning accuracy.

P	True	LassoSum	TlpSum	ElNet
.001	27.7	19.8 (3.3)	18.4 (3.1)	20.0 (3.3)
.01	302.4	109.7 (7.4)	109.3 (7.7)	109.6 (7.8)
.1	2995.7	1686.3 (299.2)	1734.6 (300)	1649 (262.3)

Table L: Table comparing the number of true nonzero effect sizes recovered by each of the penalizezd regression methods applied to simulation 1. The standard deviation across the 20 simulations is in parentheses. The number of true positives corresponds to the model with the best tuning accuracy.

P	True	LassoSum	TlpSum	ElNet
.0005	30.6	21.2 (3.8)	19.7 (4.3)	21.4 (4.0)
.001	61.8	35.7 (5.7)	34.1 (6.1)	37.3 (6.3)
.01	617.4	156.0 (17.3)	159.1 (11.3)	139.6 (7.8)
.1	6128.8	3,194.7 (938.7)	4,084.7 (890.0)	4,536.7 (507.0)

Table M: Table comparing the number of true nonzero effect sizes recovered by each of the penalized regression methods applied to simulation 2. The standard deviation across the 20 simulations is in parentheses. The number of true positives corresponds to the model with the best tuning accuracy.

P	True	LassoSum	TlpSum	ElNet
.0005	119.6	53.4 (4.4)	49.7 (6.9)	52.4 (4.1)
.001	233.1	71.8 (9.2)	68.8 (11.1)	70.1 (9.0)
.01	2312	205 (125.1)	202.8 (139.3)	229.8 (230.5)

Table N: Table comparing the number of true nonzero effect sizes recovered by each of the penalized regression methods applied to simulation 3. The standard deviation across the 20 simulations is in parentheses. The number of true positives corresponds to the model with the best tuning accuracy.

P	True	LassoSum	TlpSum	ElNet
.001	29,701	82.9 (82.2)	58.5 (17.5)	115.9 (116.9)
.01	29,475	521.6 (46.1)	521.9 (47.5)	488.1 (53.8)
.1	26,782	12,421 (3082)	12,911 (3128)	11,991 (2630)

Table O: Table comparing the number of false positive nonzero effect sizes for each of the penalizezd regression methods applied to simulation 1. The standard deviation across the 20 simulations is in parentheses. The number of false positives corresponds to the model with the best tuning accuracy.

P	True	LassoSum	TlpSum	ElNet
.0005	61,124	103.6 (15.4)	70.3 (44.1)	166.3 (82.9)
.001	61,093	114.6 (13.8)	173.1 (234.6)	222.2 (79.2)
.01	60,538	1014.0 (217.9)	1063.5 (114.8)	669.4 (80.7)
.1	55,026	32,309 (9497)	34,040 (9064)	38,532.6 (5,157 .3)

Table P: Table comparing the number of false positive nonzero effect sizes for each of the penalized regression methods applied to simulation 2. The standard deviation across the 20 simulations is in parentheses. The number of false positives corresponds to the model with the best tuning accuracy.

P	True	LassoSum	TlpSum	ElNet
.0005	229,156	328.3 (94.5)	272.4 (135.8)	261.2 (91.2)
.001	229,043	362.1 (74.6)	332.8 (110.1)	313.2 (86.3)
.01	226,965	4610 (5658)	4669 (6510)	7087 (15,867)

Table Q: Table comparing the number of false positive nonzero effect sizes for each of the penalized regression methods applied to simulation 3. The standard deviation across the 20 simulations is in parentheses. The number of false positives corresponds to the model with the best tuning accuracy.

Following are tables corresponding to the results for the simulation study for model selection. Additionally included is Fig B, which demonstrates the performance of model selection and quasi-correlation for model fit applied to TlpSum. The results are similar to the application to LassoSum.

P	Tuning R2	Pseudo AIC	True AIC	Pseudo BIC	True BIC	pseudoVal	quasiCor
.001	.435 (.025)	.421 (.023)	.427 (.023)	.410 (.026)	.427 (.027)	.427 (.023)	.434 (.024)
.01	.297 (.023)	.290 (.026)	.298 (.023)	.229 (.031)	.274 (.028)	.255 (.015)	.297 (.023)
.1	.134 (.011)	.125 (.013)	..125 (.013)	.125 (.013)	.125 (.013)	.125 (.013)	.135 (.012)

Table R: This table displays the results for LassoSum. Each cell contains the accuracy of the selected model on the testing data for the seven different methods of model selection applied to simulation 1. Accuracy is measured by predictive r^2 on the testing data. Each value is the mean across the 20 different simulation settings, with the standard deviation in parentheses.

P	Tuning R2	Pseudo AIC	True AIC	Pseudo BIC	True BIC	pseudoVal	quasiCor
.001	.439 (.025)	.420 (.023)	.435 (.028)	.417 (.027)	.428 (.030)	.426 (.024)	.436 (.026)
.01	.297 (.024)	.290 (.026)	.294 (.026)	.229 (.031)	.240 (.033)	.255 (.015)	.296 (.023)
.1	.136 (.010)	.125 (.013)	.125 (.013)	.125 (.013)	.125 (.013)	.125 (.013)	.136 (.012)

Table S: This table displays the results for TlpSum. Each cell contains the accuracy of the selected model on the testing data for the seven different methods of model selection applied to simulation 1. Accuracy is measured by predictive r^2 on the testing data. Each value is the mean across the 20 different simulation settings, with the standard deviation in parentheses.

P	Tuning R2	Pseudo AIC	True AIC	Pseudo BIC	True BIC	pseudoVal	quasiCor
.001	.461 (.035)	.452 (.039)	.458 (.035)	.431 (.037)	.450 (.033)	.459 (.037)	.461 (.035)
.01	.315 (.026)	.308 (.027)	.316 (.025)	.239 (.031)	.288 (.029)	.275 (.018)	.315 (.024)
.1	.141 (.015)	.131 (.016)	.131 (.016)	.131 (.016)	.131 (.016)	.131 (.016)	.142 (.016)

Table T: This table displays the results for LassoSum. Each cell displays the accuracy of the selected model on the testing data, as measured by the squared quasi-correlation, for the seven different methods of model selection applied to simulation 1. Each value is the mean across the 20 different simulation settings, with the standard deviation in parentheses.

P	Tuning R2	Pseudo AIC	True AIC	Pseudo BIC	True BIC	pseudoVal	quasiCor
.001	.461 (.035)	.452 (.039)	.458 (.035)	.431 (.037)	.450 (.033)	.459 (.037)	.461 (.035)
.01	.314 (.023)	.308 (.027)	.310 (.031)	.240 (.031)	.248 (.033)	.275 (.018)	.315 (.024)
.1	.148 (.014)	.136 (.016)	.136 (.016)	.136 (.016)	.136 (.016)	.136 (.015)	.149 (.015)

Table U: This table displays the results for TlpSum. Each cell displays the accuracy of the selected model on the testing data, as measured by the square of the quasi-correlation, for the seven different methods of model selection applied to simulation 1. Each value is the mean across the 20 different simulation settings, with the standard deviation in parentheses.

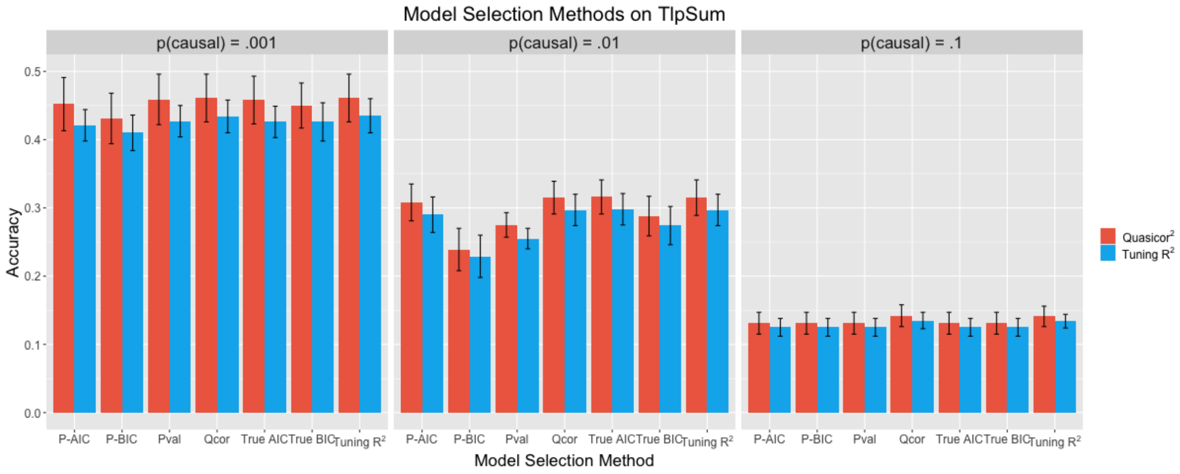

Fig B: Performance of the seven different model selection methods applied to a set of candidate TlpSum models for simulation 1. Performance is measured by r^2 on the testing data (the right bar in each group), and by squared quasi-correlation on the testing data (the left bar in each group). Error bars represent the standard deviation across 20 replications.

C Simulation Study without LD Blocks

Here, we present the predictive accuracy for the penalized regression models described in the simulation study for penalized regression estimated without LD blocks. Otherwise, the simulation settings were the same as those described in the simulation study for model selection without allelic heterogeneity, with the exception that we did not exclude SNPs with $MAF < .01$ in this simulation. Because we do not expect SNPs with $MAF < .01$ to contribute substantially to the genetic heritability of the simulated phenotype, this shouldn't have made a significant difference in estimating the predictive power of the models. The predicted accuracy for the four methods that don't use penalized regression are the same as in the results section. We find that predictive accuracy is improved modestly by implementing LD blocks in most simulation settings. Tables S22 and S23 below can be compared to tables S1 and S2 to see the differences in predictive accuracy.

P	LDPred	LDP-Inf	LassoSum	TlpSum	ElNet	PRS	PRS P+T
.001	.411 (.030)	.125 (.020)	.433 (.021)	.433 (.020)	.432 (.018)	.123 (.022)	.380 (.024)
.01	.264 (.023)	.123 (.011)	.279 (.016)	.277 (.017)	.280 (.017)	.122 (.014)	.251 (.018)
.1	.132 (.012)	.123 (.011)	.128 (.014)	.129 (.015)	.126 (.015)	.123 (.012)	.123 (.012)

Table V: Prediction r^2 values for simulation 1 estimated without LD blocks. Standard deviation across the 20 replications in parentheses.

P	LDPred	LDP-Inf	LassoSum	TlpSum	ElNet	PRS	PRS P+T
.0005	.437 (.021)	.072 (.023)	.426 (.021)	.425 (.022)	.423 (.019)	.071 (.022)	.358 (.018)
.001	.412 (.025)	.078 (.016)	.392 (.024)	.389 (.022)	.392 (.024)	.075 (.017)	.346 (.032)
.01	.219 (.015)	.076 (.009)	.187 (.016)	.180 (.013)	.187 (.015)	.074 (.009)	.161 (.103)
.1	.076 (.010)	.072 (.009)	.070 (.009)	.070 (.010)	.0327 (.005)	.072 (.010)	.072 (.010)

Table W: Prediction r^2 values for simulation 2. Standard deviation across the 20 replications in parentheses. LDPred is the most accurate in all scenarios.

Following is the table describing the number of nonzero effect size estimates for the three penalized estimation methods when estimation was done without LD blocks. Compared to table S9, we see that the penalized regression methods estimate more nonzero effect sizes when $p = .001$.

P	True	LassoSum	TlpSum	ElNet
.001	39.5	121.5	103.5	129.4
.01	416.3	569.8	696.3	464.4
.1	4144.9	14,458.1	16.487.5	12,973.3

Table X: Comparing the mean number of nonzero effect size estimates of the penalized regression methods against the ground truth in simulation 1, estimated without LD blocks. For the penalized regression estimates, the number of nonzero effect size estimates corresponds to the model with the best tuning accuracy.

In total, the increases in accuracy and model sparsity that result from implementing blockwise estimation are arguments for the use of LD blocks in the estimation of penalized regression models. Additionally, the computation time is drastically decreased by implementing LD blocks. Estimation a set of ~ 40 TLP models for $\sim 60,000$ SNPs in simulation 2 could take as long as five hours without LD blocks, while taking an average time of 15 minutes with LD blocks.

D Accuracy of Summary Statistic Approximations

In this section, we present results demonstrating the accuracy of the summary statistic based approximations that comprise the estimation of the pseudo AIC, pseudo BIC, and quasi-correlation. Given that these approximations are not perfect, we are interested in determining where the sources of error come from. This section serves to demonstrate the accuracy of the various summary statistic approximations, and how that accuracy changes depending on the simulation setting. We conclude generally that the approximations are appropriate.

D.1 Estimating Phenotypic Out-of-Sample Variance

Estimating the quasi-correlation requires estimating the phenotypic variance of the out-of-sample testing data. We show that, for the three simulation settings with different proportions of causal SNPs as described in this section, our methodology for estimating the variance of the out-of-sample phenotype as described in the methods section is highly accurate. Note that each SNP generates an estimate of out of sample variance; we used the median of the p estimates.

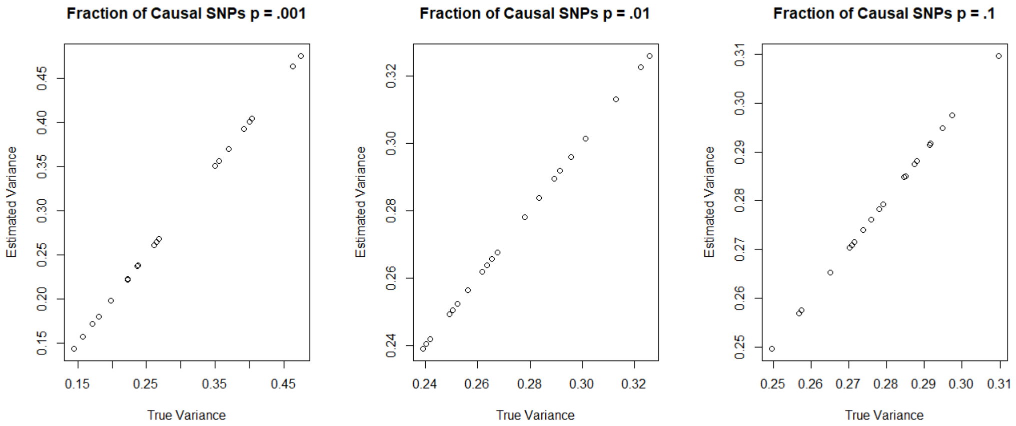

Fig C: Plot of the estimated variance versus the true variance for each of the three simulation settings. Each data point represents one of the twenty different simulations. The Pearson correlation coefficient $r = 1$ for each of these three plots.

D.2 Accuracy of Residual Variance Estimation

An important component of our pseudo AIC / BIC methods is the estimation of the residual variance $\tilde{\sigma}^2$. We present here the accuracy of the residual variance estimation for the three simulation scenarios described in this section Note that, when estimating $\tilde{\sigma}^2$, we regularize the estimated covariance matrix as described in the methods section. We select the set of SNPs used to estimate the residual variance as follows for the three scenarios. For the scenario where the probability of a SNP being causal is $p = .1$, we do clumping and pruning such that only SNPs with marginal p-value $< .01$ are included, and no two SNPs are in LD $r^2 > .2$. When the proportion of causal SNPs is $p = .01$, we do clumping and pruning with a marginal p-value cutoff of $< .001$ and an LD cutoff of $r^2 > .2$. Likewise, when the proportion of causal SNPs is $p = .001$, we have a marginal p-value cutoff of $< 1 \times 10^{-4}$ and an LD cutoff of $r^2 > .2$. Note that our method estimates the proportion of residual variance that is not explained by ordinary least squares linear regression. This is an application where OLSE will fail to capture much of the heritable variance, because of the sparse signal and nuanced correlation structure of the data. This means that our estimates $\hat{\tilde{\sigma}}^2$ are biased upwards. Nevertheless, we show that the estimates $\hat{\tilde{\sigma}}^2$ are well correlated with the true residual variance, which is known in this simulation setting.

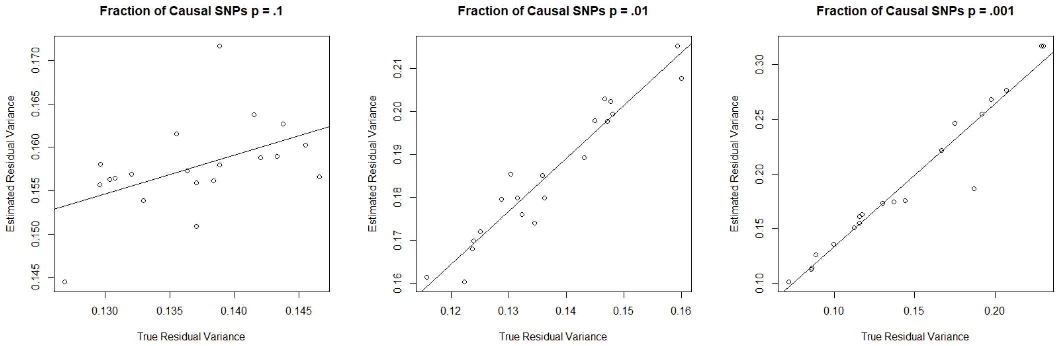

Fig D: Plot of the estimated residual variance versus the true residual variance for 20 replications at each of the three simulation settings. Note that the residual variance is overestimated in all three simulation settings.

We see that the residual variance is significantly easier to model when the fraction of causal SNPs is smaller. When the proportion of causal SNPs is $p = .001$, we have a correlation between true and estimated residual variances of $r = .97$. Likewise, when the proportion of causal SNPs is $p = .01$, we have a correlation of $r = .96$. When the proportion of causal SNPs is $p = .1$, we have a correlation of $r = .49$. To see why,

consider that estimation of $\hat{\hat{\sigma}}^2$ can be thought of as prediction, where we are using ordinary least squares estimates to predict the phenotype. As demonstrated in Fig 1, 2 and 3, prediction is more difficult as the proportion of causal SNPs p increases. Likewise, estimation of $\hat{\hat{\sigma}}^2$ is more difficult as p decreases. A further analysis of methods of residual variance estimation is in **Section E in S1 Text**.

D.3 Accuracy of SSE estimation

Another component of our pseudo AIC / BIC methods is the estimation of model SSE on the training data, as described in equation (20). For this method, we regularize the estimated covariance matrix as described in the methods section. The estimation of the residual variance involves three approximations: the approximation of $\frac{1}{n}\widehat{\mathbf{X}^T\mathbf{X}}$ (6), the approximation of $\frac{1}{N}\widehat{\mathbf{Y}'\mathbf{Y}}$ (7), and the approximation of $\frac{1}{N}\widehat{\mathbf{X}'\mathbf{Y}}$ (8). We find that the approximation described by equation (7) is nearly always accurate, as demonstrated in **Section D in S1 Text**. Likewise, the approximation described in equation (8) is very accurate. The issue arises in the approximation described in equation (6). $\frac{1}{n}\widehat{\mathbf{X}^T\mathbf{X}}$ is used to estimate the variance of the predicted phenotype in the unseen training data. It is difficult to estimate this variance, because there may be overfitting effects that are difficult to account for by using reference panel data to estimate covariance, especially for under-penalized models with a large proportion of active parameters. We offset this somewhat with the penalty described in the methods section, but it is difficult to account for completely.

The phenomenon described above explains the difference between the estimated and true SSE values. We present three different plots for each different proportion of causal SNPs. Each plot represents one of the twenty replications. Each point on the plot represents a unique TLP model, estimated via our TlpSum method.

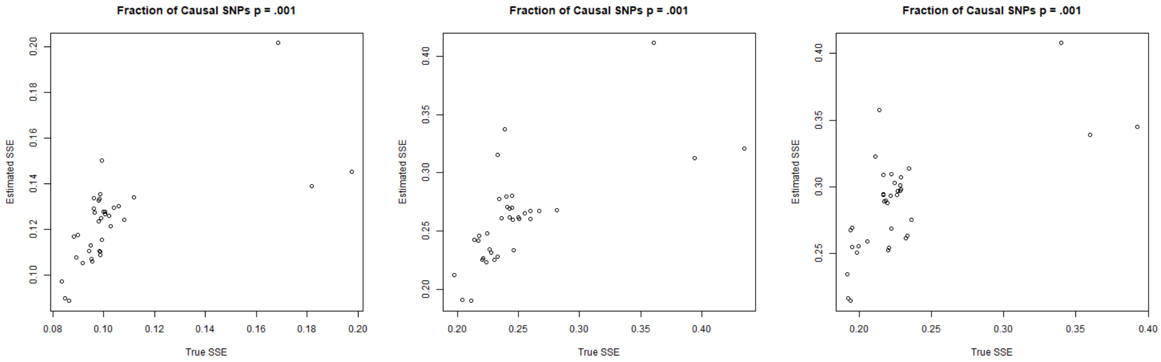

Fig E: True versus estimated SSE for TlpSum models applied to the simulation setting with the fraction of causal SNPs $p = .001$. These plots describe the relationship between the true and estimated SSE for three randomly chosen simulation settings among the twenty we conducted. Each plotted point represents one of 36 candidate models. The estimated Pearson correlation coefficients for the plots were, from left to right: $r = .65$, $r = .69$, $r = .67$

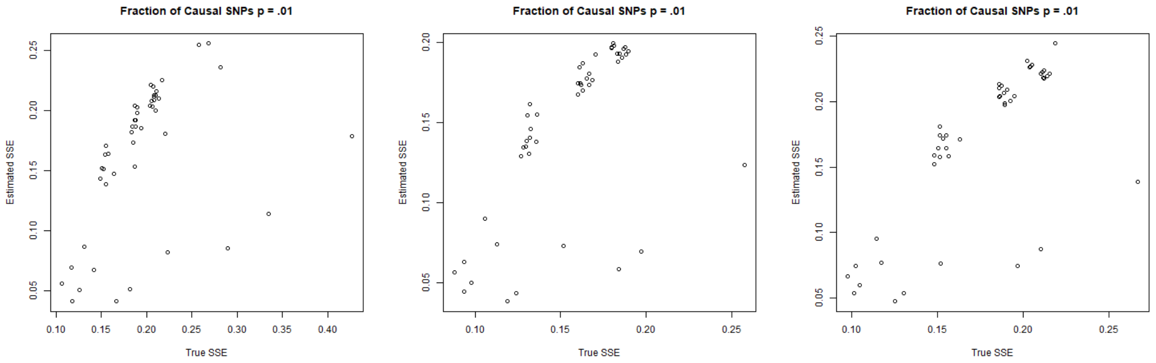

Fig F: True versus estimated SSE for TlpSum models applied to the simulation setting with the fraction of causal SNPs $p = .01$. These plots describe the relationship between the true and estimated SSE for three randomly chosen simulation settings among the twenty we conducted. Each plotted point represents one of 48 candidate models. The estimated Pearson correlation coefficients for the plots were, from left to right: $r = .41$, $r = .61$, $r = .73$

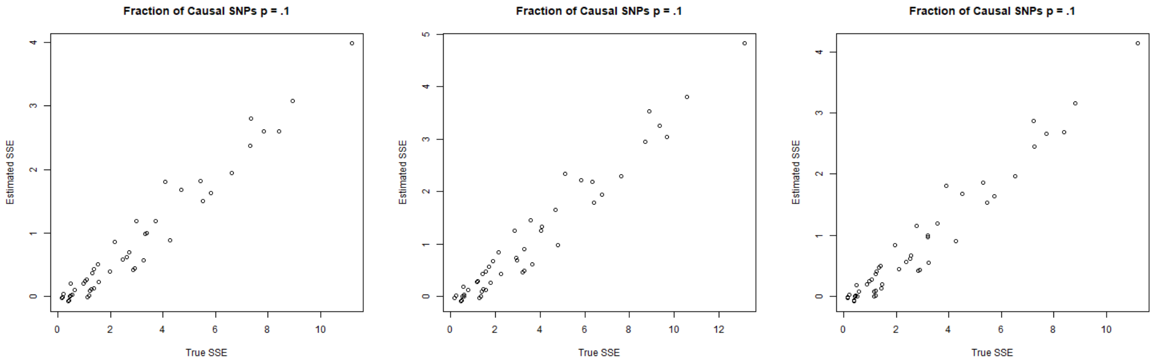

Fig G: True versus estimated SSE for TlpSum models applied to the simulation setting with the fraction of causal SNPs $p = .1$. These plots describe the relationship between the true and estimated SSE for three randomly chosen simulation settings among the twenty we conducted. Each plotted point represents one of 48 candidate models. The estimated Pearson correlation coefficients for the plots were, from left to right: $r = .98$, $r = .98$, $r = .98$

We see, contrary to the behavior of the estimated residual variance described in **Section D in S1 Text**, that the SSE is easier to estimate as the fraction of causal SNPs p increases. To demonstrate why, consider that SSE/σ^2 is distributed χ^2_{n-k}, where k is the number of active parameters in the model. As the fraction of causal SNPs p decreases, the number of active parameters in a given model k will decrease, meaning that the variance of $SSE/\sigma^2 \sim \chi^2_{n-k}$ will increase, making estimation more difficult. Most important is the behavior in the bottom left corner of these plots: we want the models with small true SSE also have small estimated SSE. By and large, we find that this is the case. We do see some systematic underestimation of the SSE. This is especially apparent by the plots for $p = .1$, where some of the estimated SSE values are in fact negative. We stress that the estimation of these SSE values is of use for model comparison especially in the context of AIC and BIC, and shouldn't necessarily be used as a reliable estimate of the magnitude of the SSE. This behavior is due to the systematic underestimation of the $\boldsymbol{\beta}'\mathbf{X}'\mathbf{X}\boldsymbol{\beta}$ term that occurs when a reference panel is used to approximate the $\mathbf{X}'\mathbf{X}$ matrix. This effect is more pronounced as the number of active parameters in the model grows.

D.4 Accuracy of Quasi-Correlation

The simulation study for model selection demonstrates that quasi-correlation does a good job approximating the true predictive r^2 on out-of-sample data for selected models. Here we expand on those results, showing

that quasi-correlation generally does a good job approximating the predictive r^2 for all candidate models.

We use our simulation 1 to investigate the performance of quasi-correlation. We investiage the performance of quasi-correlation as follows. For each of the twenty simulation settings, we have a set of candidate models. For each candidate model, we calculate the predictive accuracy on the testing data using predictive r^2, which requires individual level data, and squared quasi-correlation, which requires only summary statistics. We then calculate the correlation between the predictive r^2 values and the squared quasi-correlation values. Thus, for each combination of simulation setting (defined by the fraction of causal SNPs) and model estimation method (i.e. TlpSum or LassoSum), we have a vector of twenty correlations. Entry i of the vector corresponds to the correlation between the predictive r^2 values and the squared quasi-correlation values for the candidate models in replication i. This information is displayed in Fig H.

The reasoning behind this investigation is as follows. More of interest than whether the quasi-correlation can precisely estimate the magnitude of the predictive r^2 is whether the quasi-correlation can reliably differentiate among the out-of-sample predictive performance for a set of candidate models. If such a differentiation can be made, we can draw conclusions about the comparative quality of different models. Generally, Fig H indicates that predictive r^2 and squared quasi-correlation are well correlated, indicating that we can sufficiently differentiate between candidate models.

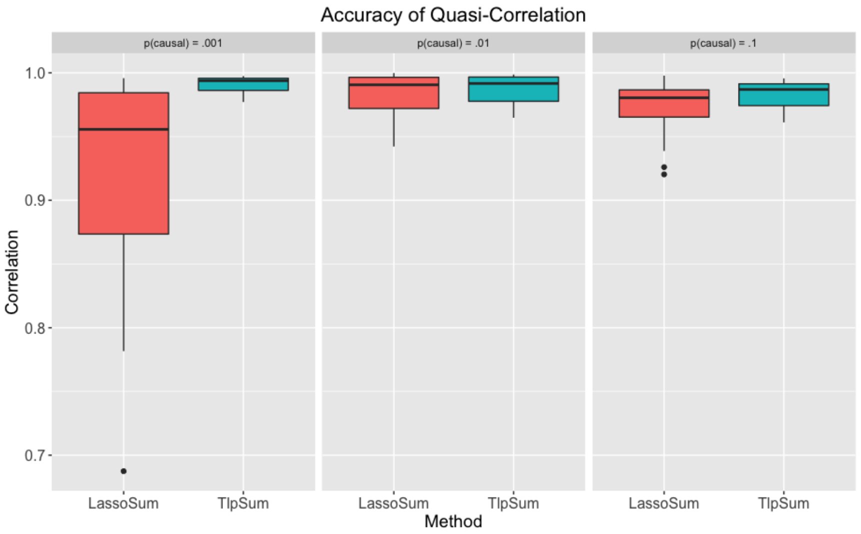

Fig H: Accuracy of quasi-correlation approximations in simulation 1. Boxplots represent distribution across twenty replications at each simulation setting of the correlation between predictive r^2 and squared quasi-correlation on out-of-sample testing data.

Fig H demonstrates that quasi-correlation approximates true correlation well in the majority of simulations. In the simulation setting with the proportion of causal SNPs $p = .001$, there are a small number of replications where the squared quasi-correlation does a somewhat poor job of approximating the predictive r^2 for the LassoSum. Nevertheless, the median value is well above .9 in all cases. Candidate sets of TlpSum models are generally well differentiated by squared quasi-correlation, as demonstrated by Fig H. Generally, the better performance of quasi-correlation in differentiating TlpSum models as opposed to LassoSum models can be explained as follows. Candidate sets of TlpSum models generally contain models with a wider spread of predictive r^2 values on out of sample data, due to the three-dimensional grid search that contains some infeasible values of τ. Quasi-correlation can differentiate quite well between models with substantially different predictive r^2 performance on out of sample data. For several replications, candidate sets of LassoSum models contain models that perform reasonably similarly on out-of-sample data. Quasi-correlation has more difficulty differentiating between these similar models, thus the correlation is lower. However, the satisfactory performance of quasi-correlation for model selection and and for assessment of model performance that we see in Fig 9 indicates that application of quasi-correlation to LassoSum models is feasible. From the results displayed here and in the simulation study for model selection, we can generally conclude that quasi-correlation is an appropriate and robust measure of predictive performance on out-of-sample data.

E Methods for Residual Variance Estimation

We investigated different methods for estimating the residual variance in high dimensional penalized regression models; that is, the expression in equation (9). Of particular interest is the so-called 'natural LASSO' estimator described in the recent paper by Yu and Bien [4]. We compared this to our methodology described in the methods section, and to other methods such as the so-called 'naive' estimator and the naive estimator with correction for model size, both also described by Yu and Bien. The estimators are defined as follows, with notation largely replicated from Yu and Bien. Consider that we have data of sample size n with p predictors, and penalized regression estimates $\hat{\beta}_\lambda$ for some tuning parameter λ. For the naive estimator, we have the following expression:

$$\hat{\sigma}^2_{naive} = \frac{1}{n}||y - X\hat{\beta}_\lambda||_2^2.$$

For the naive estimator with correction for model size, so-called $\hat{\sigma}^2_R$, we have the following expression:

$$\hat{\sigma}^2_R = \frac{1}{n - \hat{s}_\lambda}||y - X\hat{\beta}_\lambda||_2^2.$$

We define $\hat{s}_\lambda = \sum_{j=1}^p I(\beta_j \neq 0)$. Note that this is only tractable for cases where $\hat{s}_\lambda < n$, which is often not the case in our applications. This limits the usefulness of $\hat{\sigma}^2_R$ substantially. For the natural LASSO estimate, we have the following expression:

$$\hat{\sigma}^2_{nat} = \frac{1}{n} min_\beta \big(||y - X\hat{\beta}||_2^2 + 2\lambda||\beta||_1\big).$$

An oracle estimate of the residual variance, for the case where $n < |S|$ where $S = \{j : \beta_j \neq 0\}$, is defined as follows:

$$\hat{\sigma}^2_{oracle} = \frac{1}{n - |S|}||y - X_S X_S^+ y||_2^2.$$

Where we define X_S as the columns of design matrix X corresponding to indices in the set S. Clearly S is unknowable in real data applications. Our estimator $\hat{\tilde{\sigma}}^2$ can be conceptualized as an approximation of the oracle estimator, where we estimate S to be those SNPs that remain after clumping and thresholding.

The above methods for residual variance estimation can be applied to both individual level data and summary statistic data. We are able to approximate the SSE for a penalized regression model using summary statistic data, as described in the methods section. Our estimator $\hat{\tilde{\sigma}}^2$ is described in terms of summary statistics in equation (6). We investigate the performance of these residual variance estimators in application to our simulation 1 without allelic heterogeneity. We consider $\hat{\sigma}^2_{naive}$, $\hat{\sigma}^2_R$, and $\hat{\sigma}^2_{nat}$ estimated both via summary statistics and individual level data, and $\hat{\tilde{\sigma}}^2$ estimated via summary statistics only. In this application, we know the true value of the residual variance σ^2, and thus can assess the accuracy of the estimators.

Because residual variance estimators $\hat{\sigma}^2_{naive}$, $\hat{\sigma}^2_R$, and $\hat{\sigma}^2_{nat}$ depend on the penalized regression estimates $\hat{\beta}_\lambda$, it is useful to investigate the degree to which the residual variance estimators vary depending on the choice of $\hat{\beta}_\lambda$. Note that our estimator $\hat{\tilde{\sigma}}^2$ uses OLSE approximations, and thus does not depend on $\hat{\beta}_\lambda$. We investigated this as follows. For each fraction of causal SNPs p in simulation 1, we randomly chose three of the 20 replications. Each unique LassoSum model (i.e. with unique tuning parameter values s and λ) corresponds to a unique estimate of residual variance. We present the residual variance for each of the estimators versus the number of nonzero parameters in the corresponding LassoSum model. We compare those results to the true value of σ^2. The following legend was used for these plots:

- $\hat{\sigma}^2_{nat}$, as estimated from summary statistics: circle.
- $\hat{\sigma}^2_{nat}$, as estimated from individual-level data: square.
- $\hat{\sigma}^2_R$, as estimated from summary statistics: triangle pointed up.
- $\hat{\sigma}^2_R$, as estimated from real data: '+'.
- $\hat{\sigma}^2_{naive}$, as estimated from summary data: 'x'.
- $\hat{\sigma}^2_{naive}$, as estimated from real data: diamond.
- $\hat{\tilde{\sigma}}^2$, as currently used in our paper: triangle pointed down. The points are also colored red.

- The true value of σ^2: square with an x through it. The points are also colored blue.

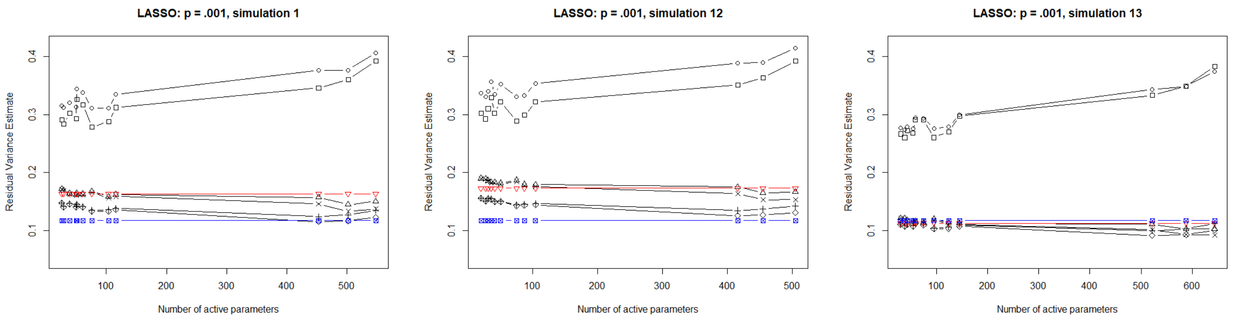

Fig I: Estimates of residual variance for simulation setting with $p = .001$, plotted against number of nonzero parameters. Estimates were calculated for each of 12 different LassoSum models.

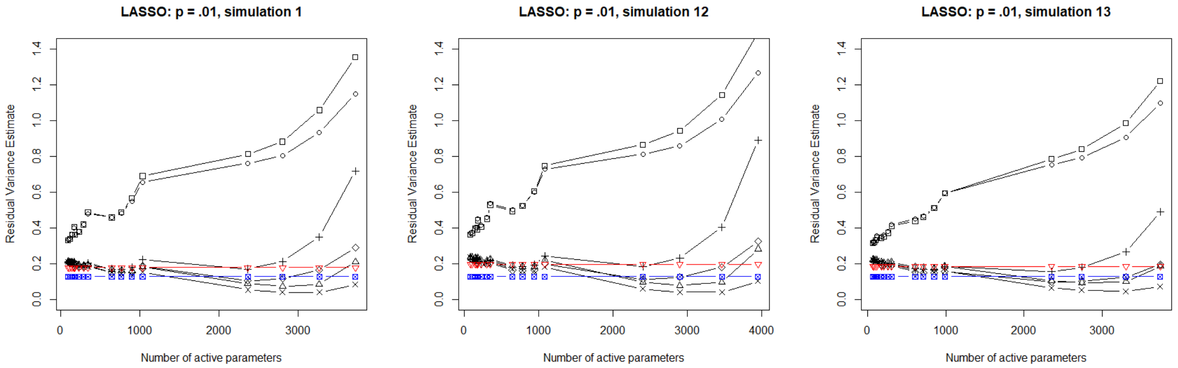

Fig J: Estimates of residual variance for simulation setting with $p = .01$, plotted against number of nonzero parameters. Estimates were calculated for 16 different LassoSum models.

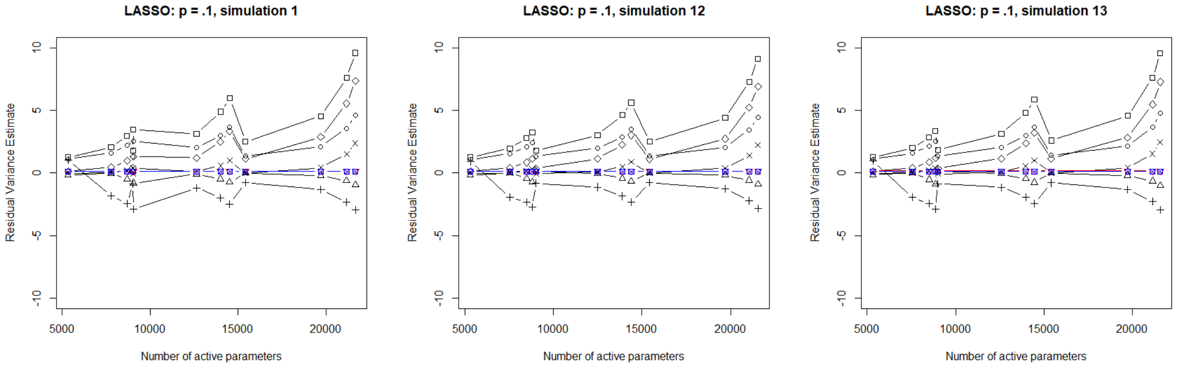

Fig K: Estimates of residual variance for simulation setting with $p = .001$, plotted against number of nonzero parameters. Estimates were calculated for 12 different LassoSum models.

When the fraction of causal SNPs $p = .001$, we see that all estimates for the residual variance perform reasonably well. There is not substantial variability in estimating σ^2 across the different LassoSum models, given that most lines are nearly horizontal. However, both the individual-level and summary statistic based estimates of $\hat{\sigma}^2_{nat}$ are substantially biased upwards. The naive estimator $\hat{\sigma}^2_{naive}$ is reasonable in simulations 1 and 12, but underestimates σ^2 in replication 13. For the construction of confidence intervals, it is generally

undesirable to underestimate σ^2, as it will lead to increased type I error. For our application to pseudo AIC/BIC, it is unclear that it is as undesirable to underestimate σ^2. We note that our estimator $\hat{\hat{\sigma}}^2$ performs relatively well in all three replications.

When the fraction of causal SNPs $p = .01$, we see increased variability depending on the LassoSum model used. Again, the natural LASSO estimates $\hat{\sigma}^2_{nat}$ are substantially biased upwards for both summary statistic based and individual based estimation, with a substantial upward trend as the number of active parameters increases. $\hat{\sigma}^2_R$ as estimated from individual level data also has a substantial upwards slope. The estimates for $\hat{\sigma}^2_{nat}$ and $\hat{\sigma}^2_R$ based on summary data show no upward trend, but do substantially underestimate the true value of σ^2 for some models. This likely relates to the behavior we notice when estimating $\widehat{SSE}$ for highly parameterized penalized regression models from summary statistic data, namely that it is difficult to approximate $\boldsymbol{\beta}^{'}\mathbf{X}^{'}\mathbf{X}\boldsymbol{\beta}$ via summary statistics. This term is often underestimated, and the effect can be substantial when the number of active parameters is large. As in the simulation setting with $p = .001$, $\hat{\hat{\sigma}}^2$ performs relatively well in all three replications.

The performance of the estimators when $p = .1$ is inconsistent. The performance of $\hat{\sigma}^2_R$ is highly unreliable and often produces negative estimate, because the denominator term $n - s_\lambda$ is often negative as the number of nonzero parameters exceeds the sample size of the training data ($n = 6240$). Likewise, the performance of the summary-statistic based $\hat{\sigma}^2_{naive}$ estimator occasionally produces negative estimates, which is troubling. This is due to the aforementioned issue, i.e. the downward bias incurred when estimating $\boldsymbol{\beta}'\mathbf{X}'\mathbf{X}\boldsymbol{\beta}$ via summary statistics. As in the other simulation settings, the natural LASSO estimates have substantial upward bias. None of the methods perform satisfactorily, with the exception of $\hat{\hat{\sigma}}^2$.

These results provide some evidence that our current method of estimating residual variance, $\hat{\hat{\sigma}}^2$, is the best option. Because $\hat{\hat{\sigma}}^2$ uses OLSE estimates, we do not incur the issue of downward bias when estimating $\boldsymbol{\beta}'\mathbf{X}'\mathbf{X}\boldsymbol{\beta}$ for overfit penalized regression models.

Another issue is the selection of a penalized regression model on which to base our estimation of σ^2, given that each different LassoSum model generates a unique estimate $\hat{\sigma}^2$. In 'On the Degrees of Freedom of the Lasso' [5], the authors suggest using the '(residual variance) estimate based on the largest model'. This seems somewhat impractical in our application, given that model size (as measured by number of active parameters) can grow to be quite large, and large models may perform quite poorly. A close analogue would be selecting the model that has the best predictive performance, i.e. the model that minimizes the corresponding estimate of $\hat{\sigma}^2$. We present the accuracy of $\hat{\sigma}^2$ estimation based on the smallest estimate for the methods discussed earlier - i.e. $\hat{\sigma}^2_{nat}$, $\hat{\sigma}^2_R$, $\hat{\sigma}^2_{naive}$, and $\hat{\hat{\sigma}}^2$ - as compared to the true value of σ^2 across 20 replications. The results are displayed below.

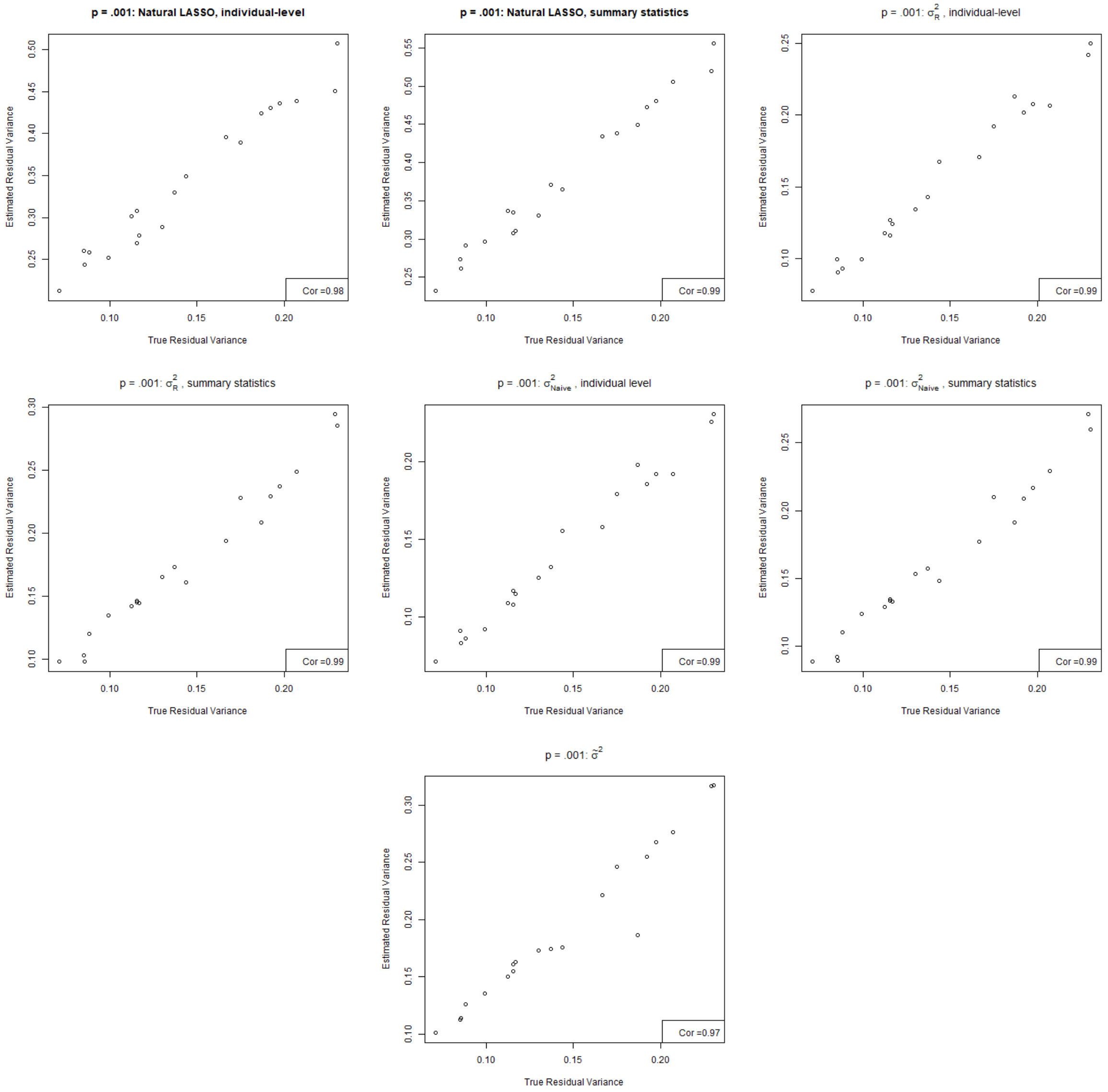

Fig L: Estimates of residual variance plotted against the true residual variance in simulation setting $p = .001$. Each point represents the estimate from one of the 20 replications.

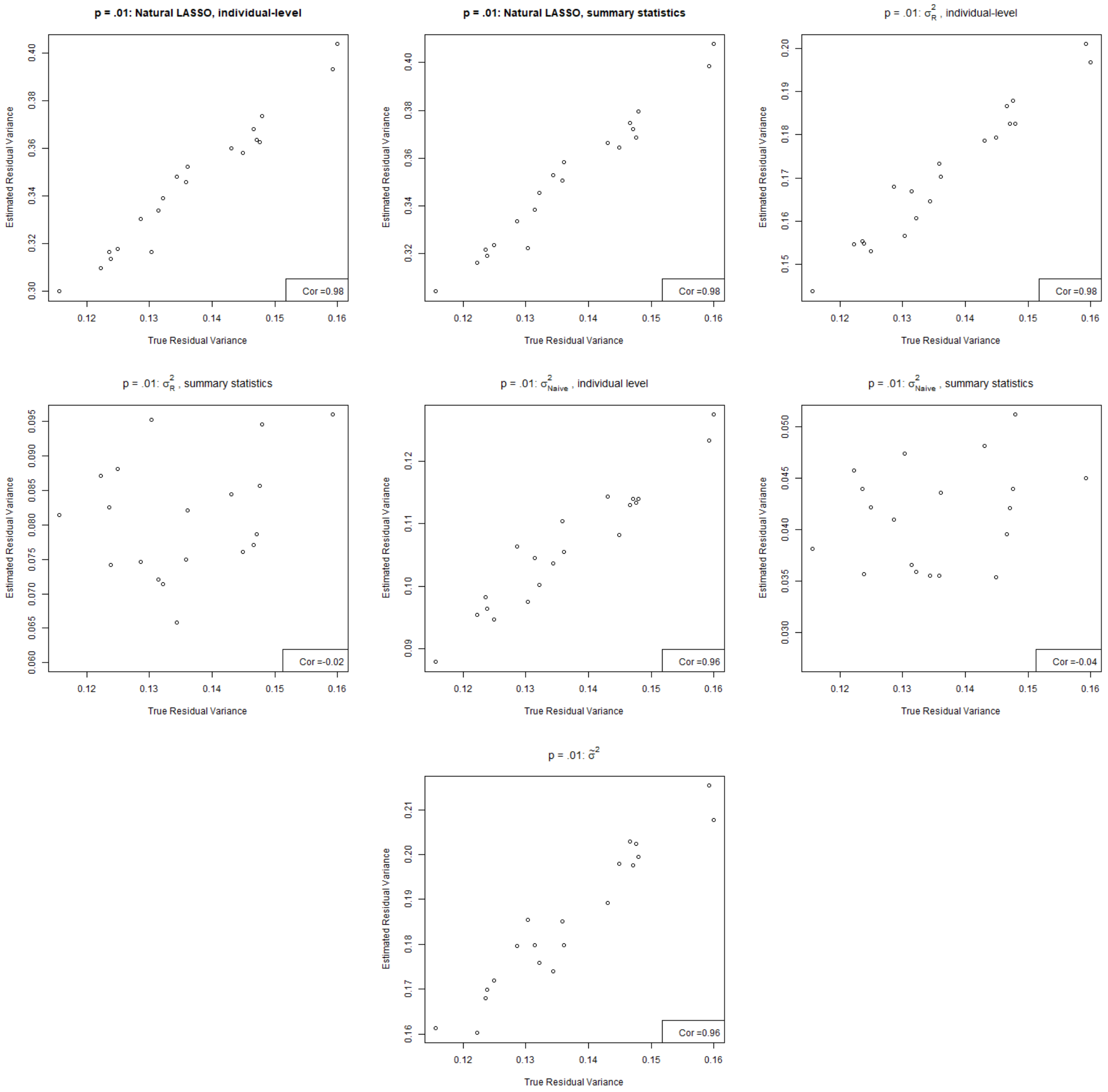

Fig M: Estimates of residual variance plotted against the true residual variance in simulation setting $p = .01$. Each point represents the estimate from one of the 20 replications.

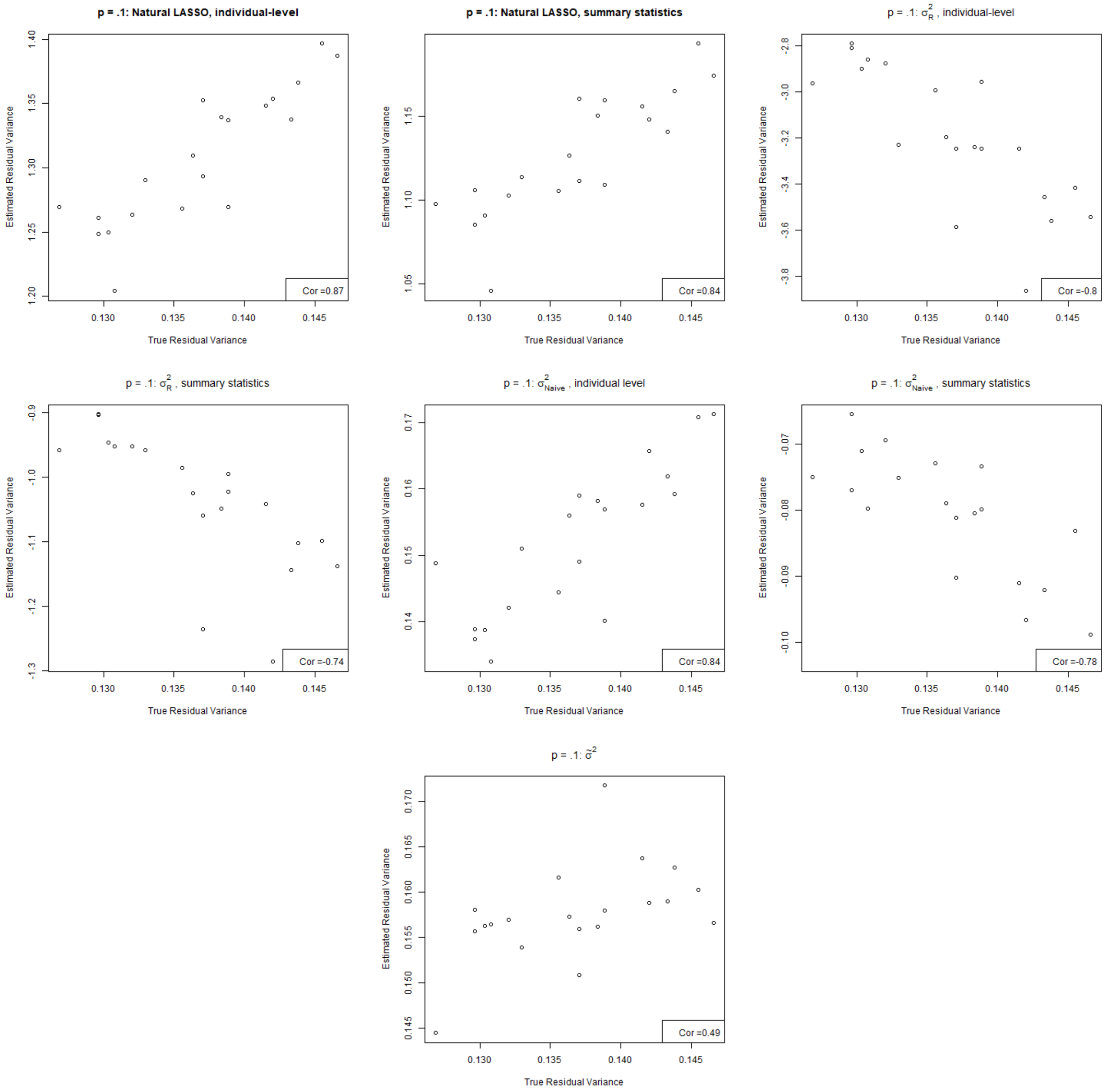

Fig N: Estimates of residual variance plotted against the true residual variance in simulation setting $p = .1$. Each point represents the estimate from one of the 20 replications.

In the simulation setting with $p = .001$, all estimates of σ^2 are well correlated with the true residual variance. The natural LASSO estimators are substantially biased upwards, while the $\hat{\sigma}^2_R$ and $\hat{\sigma}^2_{naive}$ do not show substantial bias. The $\hat{\tilde{\sigma}}^2$ have the lowest correlation with the true values of σ^2 among all of the estimators; the difference is not substantial, given that all estimates are correlated with $r > .97$. The estimates of $\hat{\tilde{\sigma}}^2$ show some upward bias, roughly commensurate with the bias shown by $\hat{\sigma}^2_R$ estimated via summary statistics.

In the simulation setting with $p = .01$, both natural LASSO estimators are well correlated with the true residual variances, although there is still substantial upward bias. The individual-level estimates of $\hat{\sigma}^2_R$ and $\hat{\sigma}^2_{naive}$ perform well, although the naive estimator shows some downward bias. The summary statistic estimations of $\hat{\sigma}^2_R$ and $\hat{\sigma}^2_{naive}$ perform quite poorly, showing both substantial downward bias and near-zero correlation with the true σ^2. This is due to the issues estimating $\boldsymbol{\beta}'\mathbf{X}'\mathbf{X}\boldsymbol{\beta}$ for overfit models, as mentioned previously. The $\hat{\tilde{\sigma}}^2$ estimates perform reasonably well, although there is some upwards bias. It appears beneficial that the $\hat{\tilde{\sigma}}^2$ avoids issues with the underestimation of $\boldsymbol{\beta}'\mathbf{X}'\mathbf{X}\boldsymbol{\beta}$ by using OLSE estimates.

In the simulation setting with $p = .1$, the performance of the estimators is fairly irregular. Both natural LASSO estimators are well correlated with the true residual variances, although there is now extreme upward bias. The summary statistic based $\hat{\sigma}^2_R$ and $\hat{\sigma}^2_{naive}$ estimators are useless. Surprisingly, the individual level

$\hat{\sigma}^2_{naive}$ performs quite well, showing low bias and strong correlation with the true σ^2. The $\hat{\hat{\sigma}}^2$ estimators perform reasonably well; they exhibit low bias, but are only moderately correlated with the true residual variances σ^2.

In total, these results indicate that our estimator $\hat{\hat{\sigma}}^2$ performs reasonably well as compared to the other methods. The natural LASSO estimators appear reasonable, although they display substantial upward bias that is undesirable in our application. The estimation of $\widehat{SSE}$ for highly parameterized penalized regression models is difficult in the context of summary statistics, which makes the performance of $\hat{\sigma}^2_R$ and $\hat{\sigma}^2_{naive}$ untenable in the simulation setting with $p = .1$ and $p = .01$. Given this, we feel that the estimator $\hat{\hat{\sigma}}^2$ is best for our application.

F Application to Lung Cancer

We apply our penalized regression methods and pseudo AIC / BIC model selection to summary statistics from a large lung cancer meta-analysis [6]. The published summary statistics contain information on 10,633 unique SNPs, all with marginal $p < 10^{-6}$. The summary statistics are drawn from meta-analyses where the sample size can vary by SNP. The distribution of the sample sizes is left-tailed, with greater than half of the summary statistics corresponding to the maximum sample size of 85,716. The smallest sample size is $\sim 10,000$.

We apply the polygenic risk scores estimated from the McKay meta-analysis to the EAGLE study. The EAGLE study, downloaded from dbGap [7], is a case-control study conducted in northern Italy, with sample size $N = 3936$. There are 1945 cases and 1991 controls. The EAGLE study was genotyped on a set of 561,466 SNPs on the Illumina HapMap550v3-B array. The data was imputed to the 1000G Phase 3 V5 reference panel using the Michigan Imputation Server [8]. After imputation, we removed all SNPs with imputation quality score $R^2 < .8$, Hardy-Weinberg p-value $< 10^{-9}$, call rate $< 90\%$, and minor allele frequency $< .01$. We were left with around 7 million SNPs.

As a baseline, we describe the performance of some polygenic risk scores that do not explicitly model linkage disequilibrium. We consider a polygenic score consisting of all marginal effect sizes from the lung cancer meta-analysis [6], which we call the full polygenic risk score. Only about half of the SNPs present in the meta-analysis achieve genome-wide significance with a marginal p-value $< 5 \times 10^{-8}$. We consider a polygenic risk score including only those SNPs that achieve genome-wide significance, the so-called genome-wide polygenic risk score. We also consider the clumped polygenic risk score. We performed clumping on the summary statistics from the McKay paper using the EAGLE data as a reference panel to calculate the correlation matrix of the SNPs. We performed clumping with PLINK [9] to prune correlated SNPs such that no two remaining SNPs are in LD $R^2 > .5$. This yielded a set of 633 SNPs. The accuracy of these methods, as compared to the penalized regression methods, is displayed in Fig O. We see that the penalized regression methods outperform the simple polygenic risk score methods. We also see that the genome-wide PRS achieves worse performance than both the simple PRS and the full PRS. This illustrates that including SNPs that are not genome-wide significant improves prediction, and that pruning based on LD helps reduce noise and improve prediction.

We also applied our penalized regression methods to construct polygenic risk scores. In line with existing practice as described in the LassoSum paper [2], we conducted LD clumping in PLINK with the densely imputed EAGLE data as a reference panel. This clumping ensured that no two SNPs had linkage disequilibrium $R^2 > .9$. Note that this clumping is significantly less stringent than the clumping used to generate the clumped polygenic risk score. After this step, there were 1524 remaining SNPs. We split the EAGLE study into three datasets; the so called tuning-1 and tuning-2 datasets, each with $N = 990$, and the test dataset with N = 1980. The tuning-1 dataset was used as a reference panel to estimate polygenic risk scores with the penalized regression methods. We estimated models without LD blocks, because of the small number of candidate SNPs. The tuning-2 dataset was used as a reference panel to estimate the model fitting criteria. The testing dataset was used to evaluate predictive accuracy.

We used the observed phenotypes from the tuning-1 data to calculate the AUC for each candidate model. We then used tuning-1 AUC as a model selection criteria, selecting the model that maximized the tuning-1 AUC. The results obtained by using tuning-1 AUC for model selection, as compared to simple polygenic score methods that don't account for LD, are displayed in Fig O. We compare the performance of tuning-1 AUC to pseudo AIC, pseudo BIC, and pseudovalidation, which do not require the existence of individual level tuning data. When calculating the pseudo AIC and pseudo BIC, we regularized the estimated covariance

matrix as described in the methods section. To facilitate the calculation of pseudo AIC / BIC for data with a binary response, we performed the method outlined in the methods section to convert the univariate logistic regression estimates to linear regression estimates. The performance of these methods, as applied to sets of candidate models generated via TlpSum and LassoSum, are displayed in Fig P.

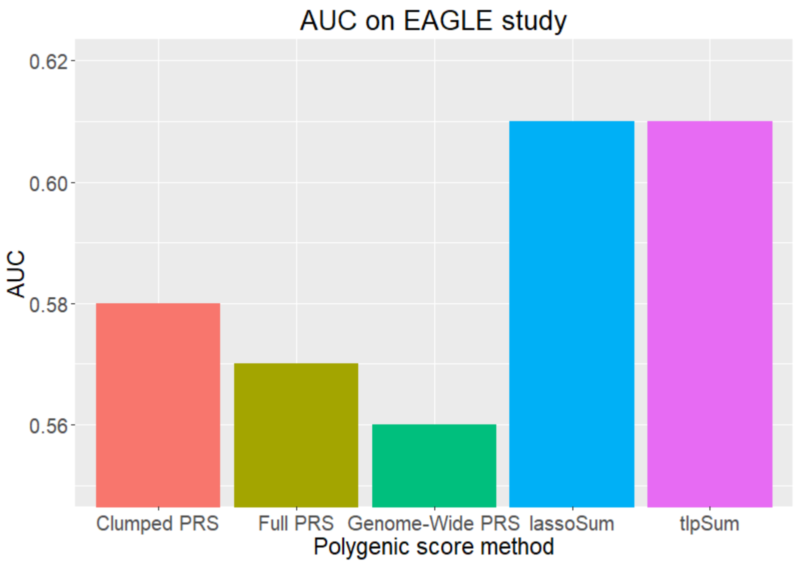

Fig O: AUC on the EAGLE study for different methods of estimating polygenic risk scores.

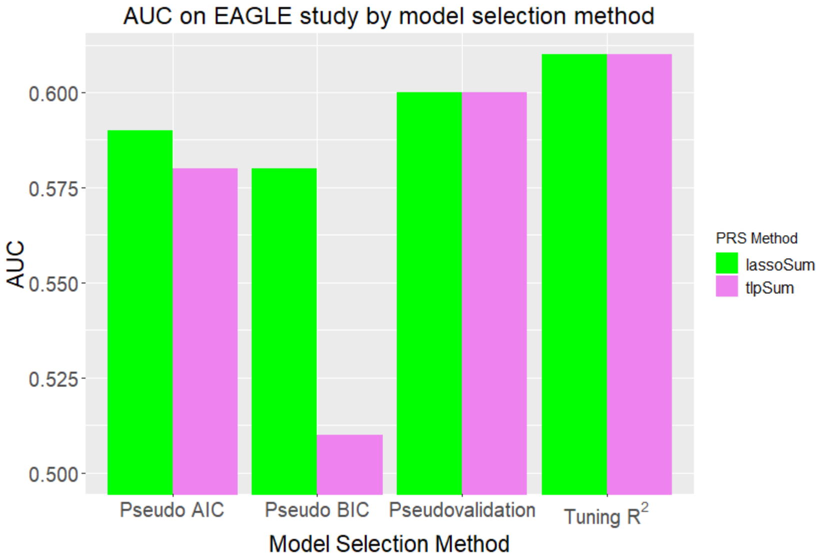

Fig P: Performance of different model selection methods applied to candidate sets of TlpSum (the left bar in each group) and LassoSum models (the right bar in each group), as measured by AUC on the testing EAGLE data.

We note, somewhat unexpectedly, that pseudovalidation is outperforming the pseudo AIC and pseudo BIC. In this application, we are only considering SNPs with a small marginal p-value, and have done pruning to ensure that no two SNPs are in LD $R^2 > .9$. Thus, it might be that most SNPs under consideration are truly associated with the phenotype. Pseudovalidation estimates predictive r^2 on the training data, which will generally select a model with many SNPs. In this scenario, where the majority of SNPs under consideration are truly associated, there may be little possibility of overfitting, and adding many SNPs to the model may be desirable. Thus, the penalties on model degree of freedom imposed by the pseudo AIC and pseudo BIC methods is likely degrading their performance. For the application to TlpSum, pseudovalidation

selects a model with 1310 nonzero parameters, which is 86.0% of the total variables considered. Pseudo AIC selects a model with 608 nonzero paramteres (39.9%), while pseudo BIC selects a model with 8 nonzero parameters (0.5%). This is further evidence that pseudo AIC and pseudo BIC impose substantial model sparsity as compared to pseudovalidation, which is shown via simulation in the results section. The increased sparsity is evidently not useful in this application.

A second issue with the application of pseudo AIC and BIC is the use of estimated SSE as a model selection metric. Our penalized regression models and our pseudo AIC / BIC methods assume a linear response when our data is binary. Although linear regression models can be estimated on data with a binary response, applying a linear regression to binary data is a model misspecification and may lead to issues. One issue is that we are using penalized SSE as our objective function in estimating the penalized regression models, and we are using SSE to estimate the pseudo AIC / BIC. A more relevant measure of model performance on binary data is AUC, which may not be closely related to SSE, as shown in the plots below. Nevertheless, our penalized regression models outperform the simple PRS methods as measured by AUC on the test data, indicating that our models have utility despite misspecifying the response. However, the plots below indicate that the use of pseudo AIC / BIC may be fraught in this case.

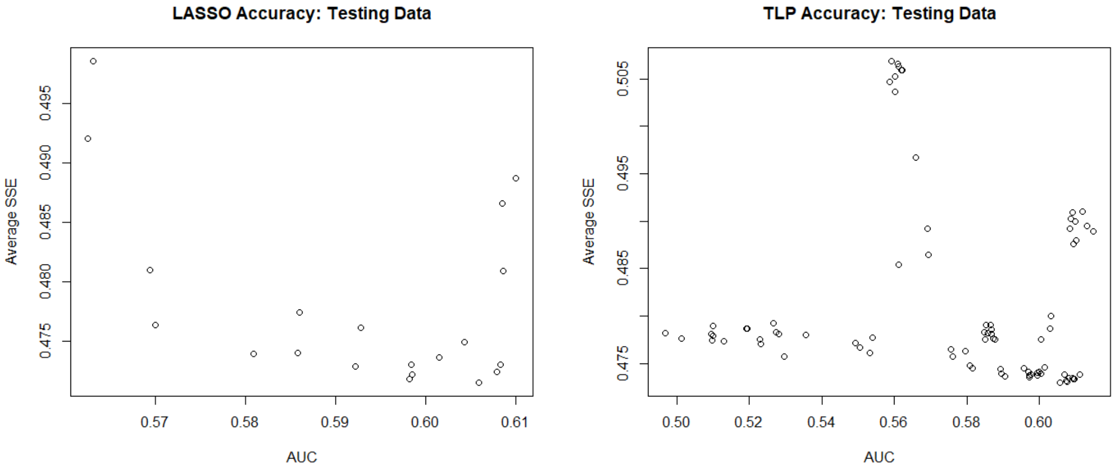

Fig Q: Plots of the performance polygenic scores estimated by LassoSum and TlpSum on the testing data. Each plotted point represents a model with a unique set of tuning parameters. The plots indicate that testing SSE and testing AUC are not associated in this case, meaning that using pseudo AIC / BIC to do model selection may be fraught.

Even given the issues with applying pseudo AIC and pseudo BIC to binary data, we view this application to lung cancer as evidence that the estimation of polygenic risk scores via penalized regression on summary statistics is useful, even when we have only a small subset of summary statistics. The penalized regression methods outperform the simple polygenic risk score and the clumped polygenic risk score, demonstrating the usefulness of accounting for linkage disequilibrium even when applied to a small subset of highly marginally significant SNPs. Pseudovalidation performs fairly well for model selection, and the pseudo AIC performs decently as well, although the performance of the pseudo BIC is quite poor for the TlpSum models. We note that lung cancer is not a strongly heritable disease, and the best models only achieve an AUC of .61. Thus, it may be difficult for pseudo AIC and pseudo BIC to select the best model, given that the difference in predictive accuracy between the candidate models is fairly small, and the signal is not very strong.

G Application to Height

We leverage our quasi-correlation and model selection methodologies to assess the fit of penalized regression models on large summary statistic data for height. We estimate polygenic risk scores on the UK BioBank data for the height, then assess the accuracy of these polygenic risk scores on the GIANT consortium data.

In this analysis, we used the UK BioBank height data [10] as our training dataset ($\sim$ 14 million SNPs, $N \sim 360,000$), 1000G data [11] as our reference panel ($\sim$ 9.5 million SNPs, $N \sim 503$) and the GIANT data

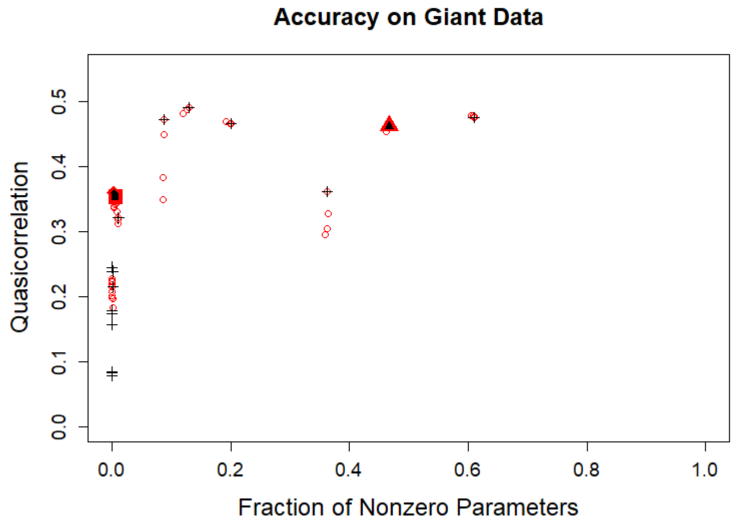

Fig R: Quasi-correlation versus fraction of nonzero parameters. The black crosses represent LassoSum models, while the red circles represent TlpSum models. Each data point represents a unique set of tuning parameters. The triangular points represent the model selected by pseudovalidation. The square points represent the model selected by pseudo AIC, while the diamond points represent the models chosen by pseudo BIC.

[12] as our testing dataset ($N \sim 130,000$, ~ 2.5 million SNPs). We limited the 1000G data to only those individuals of European ancestry. We limited the data to include only the subset of SNPs contained in all three studies. From this subset, we excluded SNPs with minor allele frequency $< .01$ in the 1000G data or the UK BioBank data. Then, we performed LD clumping using the 1000G data for LD information and the p-values from the UK BioBank study. The clumping was not especially stringent, only ensuring that no two SNPs with $R^2 > .9$ were included. From these, we also pruned out ambiguous SNPs (A/T, C/G). This left us with a set of $\sim 715,000$ SNPs.

With these SNPs, we constructed a set of candidate polygenic risk scores using TlpSum and LassoSum. We then performed model selection using our pseudo AIC and pseudo BIC methods, and compared these results to the existing pseudovalidation method for model selection [2]. We split the 1000G data into two datasets, so called 1000G-1 and 1000G-2, with $N = 252$ and $N = 251$, respectively. 1000G-1 was used as a reference panel to estimate polygenic risk scores via TlpSum and LassoSum. 1000G-2 was used as a reference panel to estimate the model fitting criteria. For the calculation of $\hat{\tilde{\sigma}}^2$ in the pseudo AIC / BIC, we used stringent clumping such that only those SNPs with marginal $p < 10^{-40}$ were leading a clump, and no two SNPs in LD $R^2 > .5$ were included. We regularized the estimated covariance matrices as described in the methods section. We present in Fig R the accuracy of the polygenic risk scores estimated via penalized regression as applied to the GIANT data.

In this application, TlpSum and LassoSum perform more or less equivalently. We see that adding more parameters to the model generally increases the predictive accuracy of the model as applied to the GIANT data. Because height is highly heritable phenotype, our training data has large sample size, and we have performed LD clumping to remove highly correlated SNPs, it is possible that many of the candidate SNPs are truly associated with the height phenotype. This means that adding more parameters to the model is generally better, which may account for the better performance of pseudovalidation as compared to pseudo AIC / BIC. The pseudo AIC / BIC select models that perform relatively well as measured by quasi-correlation, and have a small proportion of active parameters. As the proportion of active parameters increases, the models achieve modest but noticeable performance gains. However, the AIC and BIC prefer the models that perform decently while having a small proportion of active parameters. This mirrors the behavior we saw in simulation.

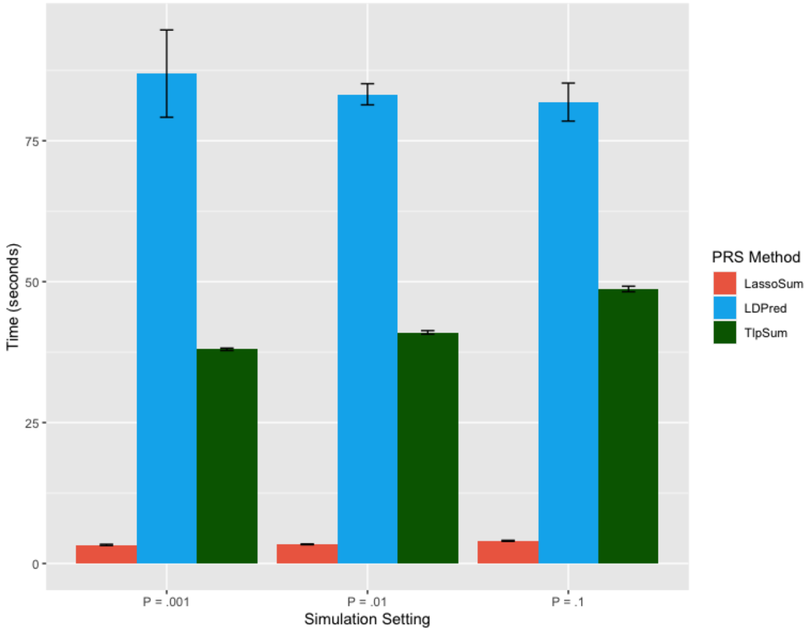

Fig S: Computational cost for three methods of estimating polygenic risk scores on summary statistics. Error bars represent standard errors across simulation replications.

H Computational Cost

We describe the computational cost of TlpSum versus LassoSum and LDPred here. Fig S shows the average computational time in seconds for a single set of tuning parameter values in our simulation 1 setting without allelic heterogeneity. LassoSum is extremely fast, while LDPred is the slowest.

I Derivation of Standard Error for Linear Regression Estimates

We justify the expression for the standard error of linear regression estimates in the methods section as follows. Let us denote the univariate logistic regression estimates as $(\hat{b}_0, \hat{b}_1)'$, and the univariate linear regression estimates as $(\hat{\beta}_0, \hat{\beta}_1)'$. Using the law of total variance and conditioning on $\hat{b}_0$, we get the expression

$$Var(\hat{\beta}_1) = Var\Big(\frac{e^{-\hat{b}_0}}{(1+e^{-\hat{b}_0})^2}\hat{b}_1\Big) = var(\hat{b}_1)E\Big(\frac{e^{-\hat{b}_0}}{(1+e^{-\hat{b}_0})^2}\Big)^2 + var\Big(\frac{e^{-\hat{b}_0}}{(1+e^{-\hat{b}_0})^2}\Big)E(\hat{b}_1)^2.$$

Plugging in $e^{-b_0} = \frac{p(Y=0)}{p(Y=1)}$ for $e^{-\hat{b}_0}$ in the first term gives us our expression derived in the methods section, namely

$$var(\hat{\beta}_1) = \Big(\frac{e^{-\hat{b}_0}}{(1+e^{-\hat{b}_0})^2}\Big)^2 var(\hat{b}_1).$$

Now, if we can show that the $var\Big(\frac{e^{-\hat{b}_0}}{(1+e^{-\hat{b}_0})^2}\Big)E(\hat{b}_1)^2$ term is negligible, our expression will be justified. To do this, consider the following. We know that $e^{-\hat{b}_0} = \frac{\hat{p}_0}{1-\hat{p}_0}$, where $\hat{p}_0 = N_{control}/N$. Thus, we can define $\frac{e^{-\hat{b}_0}}{(1+e^{-\hat{b}_0})^2} = \hat{p}_0(1-\hat{p}_0)$. We now need an expression for the variance of $\hat{p}_0(1-\hat{p}_0)$. We apply the delta method for that purpose, with the function $g(p_0) = p_0 * (1-p_0)$ and the distributional assumption that $\hat{p}_0 \sim N(p_0, \frac{p_0(1-p_0)}{N})$. Thus, we have the following expression:

$$\hat{p}_0(1-\hat{p}_0) \sim N(p_0(1-p_0), \frac{p_0(1-p_0)(1-2p_0)^2}{N})$$

Given this, we can state the following:

$$var\Big(\frac{e^{-\hat{b}_0}}{(1+e^{-\hat{b}_0})^2}\Big)E(\hat{b}_1)^2 = \frac{\hat{p}_0(1-\hat{p}_0)(1-2\hat{p}_0)^2}{N}\hat{b}_1^2$$

This term is negligible in GWAS applications. Given the small effect size of individual SNPs (and thus small $\hat{b}_1$) and the factor of N in the denominator which will be large in GWAS applications, this is reasonable. Thus, we exclude this term in our expression in the methods section.

As an additional note, we only require the use of $Var(\hat{\beta}_1)$ for the estimation of $\frac{1}{N}\widehat{\mathbf{Y}'\mathbf{Y}}$ in our equation (7). An intuitive approach, the so-called 'marginal approach' in that it is derived from the marginal distribution of $\mathbf{Y}$, is detailed as follows. Note that if $\mathbf{Y}$ is a centered binary phenotype, $\frac{1}{N}\widehat{\mathbf{Y}'\mathbf{Y}}$ is simply the variance of $\mathbf{Y}$. This is simply the variance of Bernoulli variable. Given observed $\hat{p} = N_{case}/N$ (i.e. the proportion of cases in the summary statistic data), we have that $\frac{1}{N}\widehat{\mathbf{Y}'\mathbf{Y}} = \hat{p}*(1-\hat{p})$. Assuming that we have the quantities N_{case}, N in our summary statistic data, this should be straightforward to calculate. There is, however, an issue when summary statistics are taken from a GWAS that included covariates. A detailed description of this issue is in **Section L in S1 Text**. For this reason, we recommend in general using the approach based on $Var(\hat{\beta}_1)$ we have detailed in the methods section, and not using the marginal approach to estimation of $\frac{1}{N}\widehat{\mathbf{Y}'\mathbf{Y}}$

J Additional Results for Application to Lipids

Here we present the results for applying model selection methods to a set of candidate LassoSum models in the application of our methodology to lipid data. This contrasts with the results section, where we used a set of candidate TlpSum models. Results are similar, with the pseudo-AIC outperforming pseudovalidation in all cases, and pseudo-BIC either performing equivalently or outperforming pseudovalidation.

Table Y: Model performance, as measured by quasi-correlation of the model predicted into the BioBank data, for each model selection method. Models were estimated via LassoSum on the Teslovich data.

	Quasi-cor	Pseudo AIC	Pseudo BIC	Pseudoval	Maximum
TG	.14	.13	.11	.11	.22
LDL	.13	.16	.14	.11	.21
HDL	.21	.21	.18	.18	.30

K Simulating Effect Sizes Under Allelic Heterogeneity

The algorithm for simulating effect sizes under allelic heterogeneity is as follows. Note that this simulation process assumes an ordering of the SNPs where nearby SNPS are in high linkage disequilibrium. We define h^2 as the SNP-based heritability of the disease (0.5 in our simulation), M as the number of SNPs, p as the fraction of causal SNPs, and q as the number of SNPs in the simulation. We considered values of $p = .005, p = .002$ and $h^2 \in [.2, .5, .6]$.

```
i = 0
while i ≤ q:
d ~ Bernoulli(p/5)
if(d == 0):
    β_i = 0
    i = i + 1
if(d == 1):
    k ~ round(unif(1, 7))
    for j ∈ [0, ..., k] β_{i+j} ~ N(0, h^2/(Mp))
    i = i + k
```

L Considerations for Summary Statistics Estimated with Covariates

It is often the case that summary statistics from published GWAS are estimated via multiple regression with non-SNP covariates such as age, PCs, etc. also included. Our expressions (6), (7), and (8) have been derived assuming single linear regression. These expressions are still valid assuming multiple regression with some very mild assumptions and some changes in interpretation, detailed here.

Say we have design matrix $\mathbf{Z}_i = (\mathbf{X}_i, \mathbf{Z})$, where $\mathbf{X}_i$ is one of p SNPs in the GWAS and $\mathbf{Z}$ is a set of other covariates (i.e. age, gender, PCs). Let us denote the genotype matrix of SNPs as $\mathbf{X}$, comprised of p columns $\mathbf{X}_1, \ldots, \mathbf{X}_p$. Say our summary statistics for the SNP effects consist of estimates from multiple linear regression:

$$\mathbf{Y} = \beta_i \mathbf{X}_i + \boldsymbol{\alpha}\mathbf{Z} + \boldsymbol{\epsilon} \tag{S.8}$$

Note that $\boldsymbol{\alpha}$ may be a vector. Model (S.8) would be estimated p times for each of the p SNPs in the GWAS, giving us a set of summary statistic estimates $(\hat{\beta}_1, \ldots, \hat{\beta}_p)'$. $\hat{\beta}_i$ from model (S.8) does not have straightforward application to our equation (8), given that equation (S.8) is derived assuming single linear regression. Consider the following models, again for some SNP i:

$$\mathbf{Y} = \boldsymbol{\gamma}\mathbf{Z} + \boldsymbol{\omega}_Y \tag{S.9}$$

$$\mathbf{X}_i = \boldsymbol{\lambda}_i \mathbf{Z} + \boldsymbol{\omega}_X \tag{S.10}$$

We define $\mathbf{Y}_e$ and $\mathbf{X}_{ie}$, representing the residuals from models (S.9) and (S.10), as $\mathbf{Y}_e = \mathbf{Y} - \hat{\boldsymbol{\gamma}}\mathbf{Z}$ and $\mathbf{X}_{ie} = \mathbf{X}_i - \hat{\boldsymbol{\lambda}}_i\mathbf{Z}$. We can then define the following model:

$$\mathbf{Y}_e = \tau_i \mathbf{X}_{ie} + \boldsymbol{\omega}_{ie} \tag{S.11}$$

It is the case that $\hat{\tau}_i = \hat{\beta}_i$. Thus, if we replace $\mathbf{Y}$ in our equations (7) and (8) with $\mathbf{Y}_e$, i.e. the phenotype data with the effect of non-SNP covariates regressed out, the expression holds. Consider that $\mathbf{X}_e = (\mathbf{X}_{1e}, \ldots, \mathbf{X}_{pe})$. If $\mathbf{X}_e \neq \mathbf{X}$, it will be difficult or impossible to estimate the covariance matrix of $\mathbf{X}_e$ from a reference panel.

It is a widely held implicit assumption of all summary statistic based estimation of polygenic risk scores on GWAS data with covariates that $\mathbf{X}_e \neq \mathbf{X}$, and we justify that assumption here. If we examine expression (S.10), we note that it is unlikely that a substantial proportion of the variance of a single SNP $\mathbf{X}_i$ is explained by covariates $\mathbf{Z}$, which tend to be multifactorial features such as principal components, or features that are uncorrelated with the SNP such as age. Thus, we make the implicit assumption that $\boldsymbol{\lambda}_i \approx 0, \forall i$. Thus, we assume that $\mathbf{X}_e = \mathbf{X}$. Existing methods, such as LassoSum, LDPred, and pseudovalidation, are widely applied to summary statistic data that was estimated using multivariable regression, i.e. with non-SNP covariates. All these methods make the implicit assumption that $\boldsymbol{\lambda}_i \approx 0, \forall i$. Thus, we do not need to replace $\mathbf{X}$ with $\mathbf{X}_e$ in our equations (6) and (8).

This has interesting application to our equation (7). As referenced in **Section H in S1 Text**, we do not recommend the use of the marginal approach to estimation of $\mathbf{Y}'\mathbf{Y}$ for binary $\mathbf{Y}$ when summary statistics are taken from a GWAS that includes non-SNP covariates. The reasoning behind this is as follows. If the covariates $\mathbf{Z}$ control a substantial proportion of the variation in $\mathbf{Y}$, then $\mathbf{Y}_e$ will have substantially different variance than $\mathbf{Y}$. We implicitly replace $\mathbf{Y}$ with $\mathbf{Y}_e$ when summary statistics are estimated with covariates, which the marginal approach to estimation of $\mathbf{Y}'\mathbf{Y}$ does not account for. Thus, estimation of $\mathbf{Y}'\mathbf{Y}$ for binary data is best done via the methodology outlined in the methods section.

References

[1] J. Friedman, T. Hastie, H. Höfling, and R. Tibshirani. "Pathwise Coordinate Optimization". In: *The Annals of Applied Statistics* 1 (2007), pp. 302–332.

[2] T.S.H. Shin, R.M Porsch, S.W. Choi, X. Zhou, and P.K. Sham. "Polygenic Scores via Penalized Regression on Summary Statistics". In: *Genetic Epidemiology* 41(6) (2017), pp. 469–480.

[3] X. Shen, W. Pan, and Y. Zhu. "Likelihood-based Selection and Sharp Parameter Estimation". In: *J Am Stat Assoc* 107(497) (2012), pp. 223–232.

[4] G Yu and J Bien. "Estimating the error variance in a high-dimensional linear model". In: *Biometrika* 106(3) (2019), pp. 533–546.

[5] H. Zou, T. Hastie, and R. Tibshirani. “On the Degrees of Freedom of the Lasso”. In: *The Annals of Statistics* 35 (5) (2007), pp. 2173–2192.

[6] J.D. McKay, R.J. Hung, X. Zong, R. Carreras-Torres, D.C. Christiani, N.E. Caporaso, M. Johansson, X. Xiao, et al. “Large-scale association analysis identifies new lung cancer susceptibility loci and heterogeneity in genetic susceptibility across histological subtypes”. In: *Nature Genetics* 49(7) (2017), pp. 1126–1132.

[7] dbGap. The data/analyses presented in the current publication are based on the use of study data downloaded from the dbGap website, under phs000093.v2.p2 `https://www.ncbi.nlm.nih.gov/projects/gap/cgi-bin/study.cgi?id=phs000093`. Accessed 2017.

[8] S. Das, L. Fofer, S. Schönherr, C. Sidore, A.E. Locke, A. Kwong, S.I. Vrieze, et al. “Next-generation genotype imputation service and methods”. In: *Nature Genetics* 48 (2016), pp. 1284–1287.

[9] S Purcell. *PLINK Version 1.9*. `http://pngu.mgh.harvard.edu/purcell/plink/`. 2018.

[10] C. Sudlow, J. Gallacher, N. Allen, V. Beral, P. Burton, J. Danesh, P. Downey, et al. “UK Biobank: An Open Access Resource for Identifying the Causes of a Wide Range of Complex Diseases of Middle and Old Age”. In: *PLOS Medicine* 12(3) (2015).

[11] The 1000 Genomes Project Consortium. “A Global Reference for Human Genetic Variation”. In: *Nature* 526 (2015), pp. 68–74.

[12] A.R. Wood, T. Esko, J. Yang, S. Vedantam, T.H. Pers, S. Gustafsson, A.Y. Chu, K. Estrada, et al. “Defining the role of common variation in the genomic and biological architecture of adult human height”. In: *Nature Genetics* 46(11) (2014), pp. 1173–86.
